# Structural Characterization of Cu(I)/Zn(II)-metallothionein-3 by Ion Mobility Mass Spectrometry and Top-Down Mass Spectrometry

**DOI:** 10.1021/acs.analchem.3c00989

**Published:** 2023-07-13

**Authors:** Manuel David Peris-Díaz, Sylwia Wu, Karolina Mosna, Ellen Liggett, Alexey Barkhanskiy, Alicja Orzeł, Perdita Barran, Artur Krężel

**Affiliations:** † Department of Chemical Biology, Faculty of Biotechnology, University of Wrocław, F. Joliot-Curie 14a, 50-383 Wrocław, Poland; ‡ Michael Barber Centre for Collaborative Mass Spectrometry, Manchester Institute of Biotechnology, 131 Princess Street, Manchester M1 7DN, United Kingdom

## Abstract

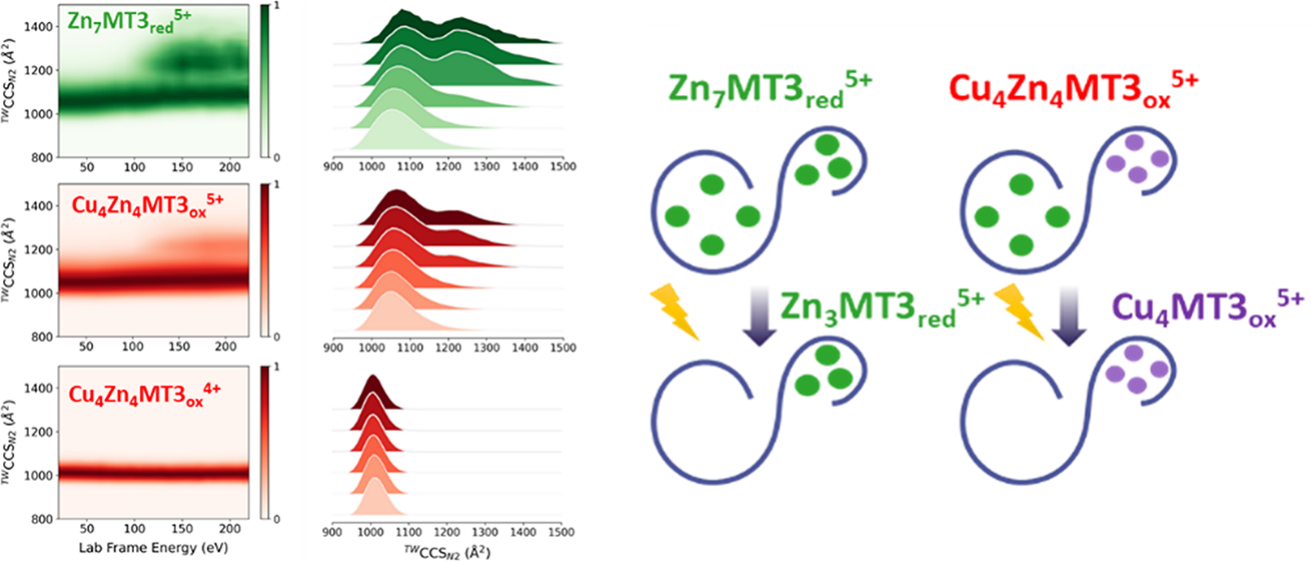

Mammalian zinc metallothionein-3 (Zn_7_MT3) plays an important role in protecting against copper toxicity by scavenging free Cu(II) ions or removing Cu(II) bound to β-amyloid and α-synuclein. While previous studies reported that Zn_7_MT3 reacts with Cu(II) ions to form Cu(I)_4_Zn(II)_4_MT3ox containing two disulfides (ox), the precise localization of the metal ions and disulfides remained unclear. Here, we undertook comprehensive structural characterization of the metal-protein complexes formed by the reaction between Zn_7_MT3 and Cu(II) ions using native ion mobility mass spectrometry (IM-MS). The complex formation mechanism was found to involve the disassembly of Zn_3_S_9_ and Zn_4_S_11_ clusters from Zn_7_MT3 and reassembly into Cu(I)_*x*_Zn(II)_*y*_MT3_ox_ complexes rather than simply Zn(II)-to-Cu(I) exchange. At neutral pH, the β-domain was shown to be capable of binding up to six Cu(I) ions to form Cu(I)_6_Zn(II)_4_MT3_ox_, although the most predominant species was the Cu(I)_4_Zn(II)_4_MT3_ox_ complex. Under acidic conditions, four Zn(II) ions dissociate, but the Cu(I)_4_-thiolate cluster remains stable, highlighting the MT3 role as a Cu(II) scavenger even at lower than the cytosolic pH. IM-derived collision cross sections (CCS) reveal that Cu(I)-to-Zn(II) swap in Zn_7_MT3 with concomitant disulfide formation induces structural compaction and a decrease in conformational heterogeneity. Collision-induced unfolding (CIU) experiments estimated that the native-like folded Cu(I)_4_Zn(II)_4_MT3_ox_ conformation is more stable than Zn_7_MT3. Native top-down MS demonstrated that the Cu(I) ions are exclusively bound to the β-domain in the Cu(I)_4_Zn(II)_4_MT3_ox_ complex as well as the two disulfides, serving as a steric constraint for the Cu(I)_4_-thiolate cluster. In conclusion, this study enhances our comprehension of the structure, stability, and dynamics of Cu(I)_*x*_Zn(II)_*y*_MT3_ox_ complexes

Mammalian metallothioneins (MTs) are small (∼6–7 kDa) proteins that are rich in cysteine residues and play a crucial role in the metabolism of zinc and copper (Zn(II) and Cu(I)).^1−3^ MTs also act as a detoxification system by binding potentially harmful metal ions such as Cd(II), Pb(II), Ag(I), or Hg(II).^4^ There are a dozen different MT proteins (MT1–MT4 isoforms and multiple subisoforms), each with distinct metal-binding properties and tissue localization.^5^ MT1 and MT2 have widespread tissue expression, while MT3 and MT4 are specifically found in the central nervous system (CNS) and stratified epithelial tissue, respectively.^6,7^ Initially identified as a neuronal growth-inhibitory factor in 1991, MT3 interacts with brain proteins and plays a role in regulating Zn(II) trafficking and controlling neurodegeneration in the CNS.^7,8^ MT3 acts as a Zn(II) sensor in the synaptic cleft, regulating synaptic exocytosis and preventing neurodegenerative disorders by swapping Zn(II) for excess copper (Cu(II)/Cu(I)) in the brain.^9^

So far, there has been only one X-ray structure solved for the hepatic rat mix Cd_5_Zn_2_MT2 species.^10^ The protein folds into a unique dumbbell-shaped structure, with the α-domain forming a Cd_4_Cys_11_ cluster and the β-domain containing the Cd_1_Zn_2_Cys_9_ cluster.^10^ In general, when divalent metal ions (M) are present, the 20 cysteine residues (21 in the case of MT1b) form metal-binding clusters, with the N-terminal β-domain forming an M_3_Cys_9_ and the C-terminal α-domain forming an M_4_Cys_11_.^5^ However, MT3 has several structural differences that may contribute to its functional differences from those of other MT isoforms. It is the only mammalian MT isoform with two prolines in its β-domain and an acidic hexapeptide loop in its α-domain.^11^ This hexapeptide loop was found to be responsible for the low stability of the metal cluster in the α-domain, likely due to the increased solvent-exposed surface area of the polypeptide.^12−14^ Additionally, unlike most MTs, MT3 expression is not induced by metal ions, suggesting a different biological role compared to other MT isoforms.^15,16^ Due to its unique structural features, such as the presence of the prolines in the conserved T_5_CPCP_9_ motif in the β-domain, MT3 displays stronger copper binding character than MT1 or MT2. As a result, it is purified from mammalian brains as a heterometallic complex, Cu(I)_4_Zn(II)_3–4_MT3.^17−20^*In vitro* studies have shown that Zn_7_MT3 can scavenge free Cu(II) ions or remove Cu(II) bound to amyloid β peptides and α-synuclein, thereby eliminating harmful redox chemistry.^21−25^ This highlights the potential role of MT3 in controlling low levels of free Cu(II) in the brain and mitigating the effects of oxidative stress, which is implicated in the pathogenesis of several neurodegenerative disorders.^26^ The mechanism of the reaction involves a Zn(II)-to-Cu(I) exchange, reducing Cu(II) to Cu(I) through the formation of two intramolecular disulfide bonds (indicated here as “ox”) (Cu(I)_4_Zn(II)_4_MT3_ox_); however, their localization has not yet been thoroughly investigated.^27^ The results of biophysical characterization of the Cu(I)_4_Zn(II)_3–4_MT3_ox_ complex showed that copper forms a Cu(I)_4_-thiolate cluster, likely located in the β-domain, which has been shown using isolated domains.^27−29^ The close proximity of the Cu(I) ions in the cluster, with distances less than 2.8 Å, allows for d^10^-d^10^ orbital overlap and provides exceptional stability and resistance to oxidation in air-exposed conditions.^28^

Mass spectrometry techniques, including nanoelectrospray mass spectrometry (nano ESI-MS) and ion mobility-mass spectrometry (IM-MS), have been effectively used to analyze heterogeneous protein systems, enabling the characterization of their conformation and dynamics.^30−35^ Meloni and Vašák measured the masses of the complexes formed upon the titration of Zn_7_MT3 with Cu(II) ions, finding that the Cu(I)_4_Zn(II)_4_MT3_ox_ should contain two disulfides.^27^ In the same work, the Cu(I)_4_-thiolate cluster was localized based on isolated domains obtained through subtilisin digestion and measured by low-temperature luminescence. Recently, Fan and Russell utilized IM-MS to analyze the Cu(I)-MT2 complexes that were formed upon the addition of Cu(I) (not Cu(II)) to metal-free MT2 (apo-MT2).^35^ Their findings revealed the existence of several species including αCu_4_MT2, βCu_6_MT2, and βCu_6_αCu_4_MT2. These results are in line with those from the Stillman group, who studied Cu(I) binding to apoMT1a and showed that the α-domain binds four Cu(I) ions, while the β-domain binds six Cu(I), forming copper-thiolate clusters.^36,37^ In their recent research, they showed that Cu(I) (not Cu(II)) binds to Zn_7_MT3 forming multiple species, with no preference for Cu_4_Zn_4_MT3.^38^ In another study, Melenbacher et al. studied Cu(I) binding to Zn_7_MT1a instead of apoMT1a.^39^ Their results suggested the formation of two main complexes, βZn_1_Cu_5_αZn_4_MT1a and βCu_6_αZn_4_MT1a. Furthermore, in the presence of excess glutathione (GSH), Austin and co-workers reported, based on ITC analysis, that Zn_7_MT2 and Zn_7_MT3 can form Cu(I)_4_-thiolate clusters, resulting in βCu_4_αCu_4_MT2_ox_ and βCu_4_αCu_4_MT3_ox_.^40^

Here, using native ion mobility mass spectrometry, we characterized the conformation and dynamics of the metal-protein complexes formed by the reaction between Zn_7_MT3_red_ (where “red” denotes reduced cysteine residues in the protein) and Cu(II) ions. Our results showed the formation of Cu(I)_4_Zn(II)_4_MT3_ox_, which contains two disulfides and has four Cu(I) ions bound to the β-domain in the full-length protein. IM-MS provides information about charge state distribution (CSD) and collision cross section (CCS).^41^ Extended conformations with larger CCS experience more collisions with the buffer gas, resulting in a longer drift time or reduced mobility. The complex was found to be smaller and less flexible than Zn_7_MT3_red_, as indicated by a lower and narrower distribution of traveling-wave-derived collision cross section (^*TW*^CCS_*N2*_) values. Gas-phase activation of protein ions via collisional activation (CA) can be used to probe subtle structural differences between similar conformations and study protein ion stability and dynamics.^42−44^ Recording IM-MS under different CA conditions showed that Cu(I)_4_Zn(II)_4_MT3_ox_ was more compact and stable compared to Zn_7_MT3_red_. The formation of Cu(I)_4_MT3_ox_ (full-length protein) upon acidification suggests that MT3 is an effective Cu(II) scavenger even at low pH. Native top-down collision-induced dissociation (CID) experiments reveal that while Zn(II) ions were partially distributed in both α- and β-domains in Zn_7_MT3_red_, the Cu(I) ions were found exclusively in the β-domain in Cu(I)_4_Zn(II)_4_MT3_ox._

## Experimental Section

We overexpressed and purified MT3 (Addgene plasmid ID 105710) in a bacterial system as described in the Supporting Information. UV–vis spectra were recorded on a JASCO V-750 spectrophotometer at 25 °C in 25 °C, in a 1 cm quartz cuvette as described in the Supporting Information.

MS and IM-MS experiments were carried out on a Synapt XS HDMS instrument equipped with nanoelectrospray ionization (Waters Corporation, Manchester, UK). 3–10 μL of sample (10–20 μM in 200 mM ammonium acetate) was loaded into borosilicate glass capillaries (O.D. 1.2 mm, I.D. 0.9 mm, World Precision Instruments, Stevenage, UK) produced in-house using a Flaming/Brown P-1000 micropipette puller (Sutter Instrument Co., Novato, CA, USA), and ions were produced by applying a positive potential of 0.9–1.4 kV via a platinum wire (Goodfellow). Two sets of TWIMS parameters of traveling wave (TW) velocity and height were used, 300 ms^–1^ and 20 V and 480 ms^–1^ and 20 V. Collision-induced unfolding (CIU) experiments were performed by increasing trap collision energies (0–60 V range) of quadrupole-selected ions and recording ion arrival time distributions. We used ubiquitin (bovine), cytochrome C (equine heart), and β-lactoglobulin (bovine milk) to calibrate the TW device.^45,46^

Native top-down collision-induced dissociation (CID) mass spectrometry experiments were performed by applying 20–60 V trap collision energies of quadrupole-selected ions with argon as the collision gas. Calculation of the survival yield curves^47,48^ and data analysis information^49^ can be found in the Supporting Information.

To perform top-down electron transfer dissociation (ETD) mass spectrometry experiments, the sample was introduced using ESI via a syringe with a 3 μL·min^–1^ flow rate and a spray voltage of 2.5 kV. The glow discharge was tuned to obtain an ETD reagent (1,4-dicyanobenzene) current of ∼1e6 counts/s for charge reduction. The anions were accumulated in the trap collision cell for 100 ms using a refill interval of 1 s. The reaction was started by lowering the wave height from 1.5 to 0.2–0.3 V, using a wave velocity of 300 ms^–1^. Data were analyzed by means MassLynx v4.2 (Waters Corp., UK), ORIGAMI, and custom scripts in Python 3.5 (available in https://github.com/ManuelPerisDiaz/Cu-Zn-MT3).^46^

## Results and Discussion

**Figure 1:**
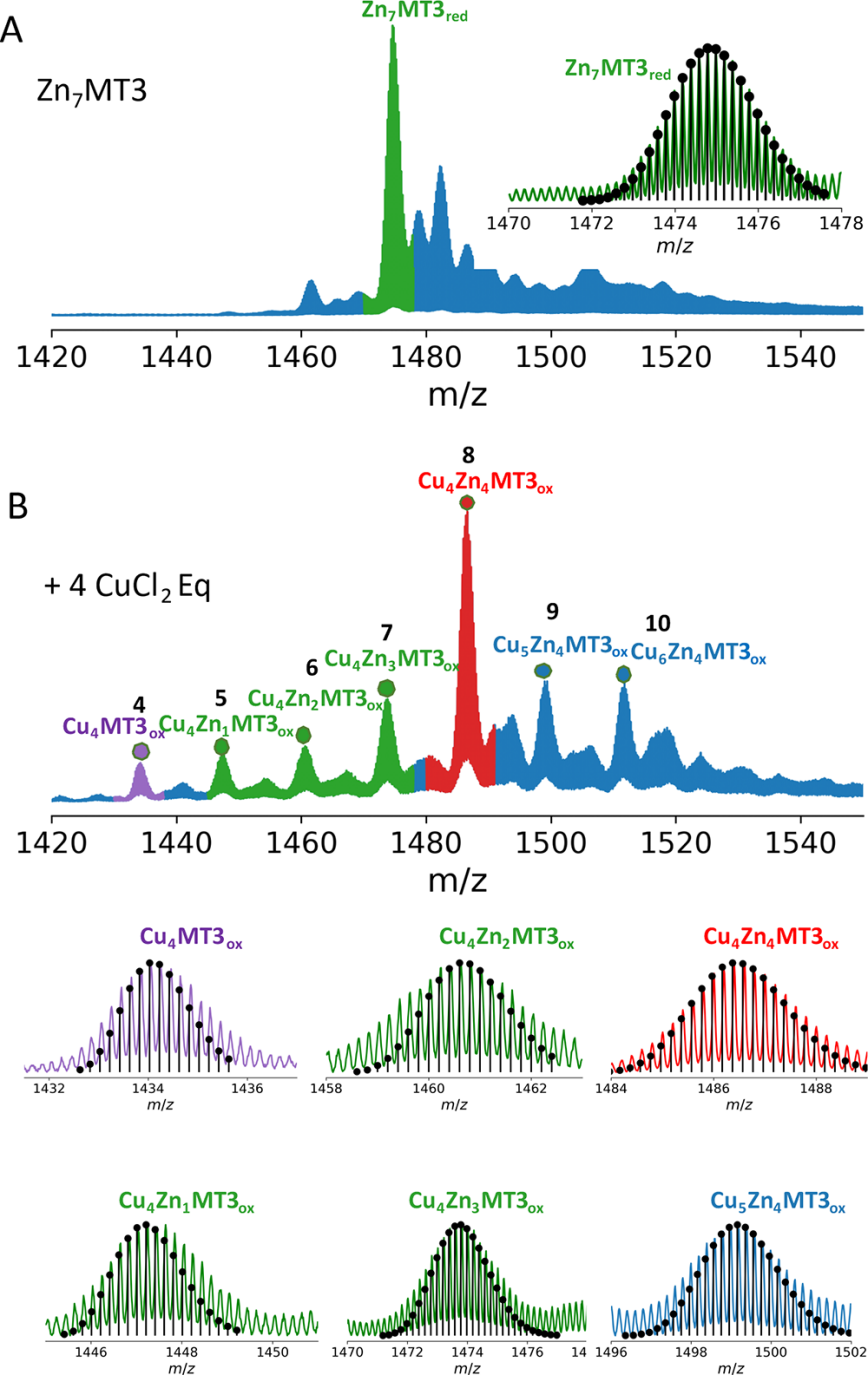
Native mass spectra of Zn_7_MT3 and the products upon addition of 4 CuCl_2_ eq (A,B). The *m*/*z* region is shown for 5+ ions. Simulations of theoretical isotopic patterns for individual proteins were plotted as stem plots. The molecular formulas can be found in Table S1. “red” and “ox” subscripts refer to reduced and oxidized (two intramolecular disulfides) MT3 proteins.

To monitor the reaction between Zn_7_MT3_red_ and Cu(II) (CuCl_2_) and the assembly of a Cu(I)_4_-thiolate cluster, we utilized isotopically resolved native mass spectrometry (MS) data. The nESI mass spectra of Zn_7_MT3_red_ sprayed with 200 mM AmAc (pH 6.8) showed a charge state distribution (CSD) covering three charge states, 3 ≤ *z* ≤ 5 (Figure S1). Upon incubating the Zn_7_MT3_red_ protein with 1 CuCl_2_ mol equivalent (eq), we observed the rapid and cooperative formation of the Cu(I)_4_Zn(II)_4_MT3_ox_ complex along with other metal-MT3 species (Figure S1). As the molar eq of CuCl_2_ was increased, new signals appeared in the mass spectrum, which temporarily overlapped with previous signals until the previous ones eventually disappeared. The assignment of metal stoichiometries in systems containing both Cu(I) and Zn(II) ions presents a challenge, as the two metals differ only by an average mass of 1.8 Da, and their isotope patterns overlap. To determine the stoichiometry accurately, multiple theoretical protein isotopic distributions were generated, and the one with the best fit was selected (Table S1). Figure S2 depicts the computational workflow for generating multiple isotopic patterns, fitting them to the experimental data, and scoring the results. Accurate mass measurements and fitting of the isotopic distributions indicated that Cu(II) is reduced to Cu(I) by oxidizing four cysteine residues from the protein and displaces Zn(II) from Zn_7_MT3_red_ to form Cu(I)_4_Zn(II)_4_MT3_ox_ with two intramolecular disulfide bonds (Figure 1A,B, Table S1). The transition from a tetrahedral Zn(II) environment to diagonal/trigonal Cu(I) coordination occurred without proton dissociation or association. All Cys residues in Zn_7_MT3_red_ were bound to seven Zn(II) and were deprotonated, and since we did not observe a change in the number of protons during the displacement of Zn(II) by Cu(I) to form the Cu(I)_4_Zn(II)_4_MT3_ox_, we infer that the noncoordinating Cu(I) residues in the Cu(I)_4_Zn(II)_4_MT3_ox_ complex have formed disulfides (Figure 1B).

We also observed all series from tetra- to heptametallic complexes, which suggest that the Cu(I)-to-Zn(II) swap proceeds by first Zn(II) cluster disassembly (Zn_3_S_9_ and Zn_4_S_11_) and then reassembly following a probability distribution (Figure 1B). We do not expect that these partially metal-loaded MT3 species are a result of gas-phase dissociation, as we could maintain seven intact Zn(II) bonds bound to Zn_7_MT3_red_ (Figure 1A). Other higher Cu(I)-loaded states were also present, in particular, Cu(I)_5_Zn(II)_4_MT3_ox_ and Cu(I)_6_Zn(II)_4_MT3_ox_ (Table S1). To shed more light on this process, we monitored the reaction of Zn_7_MT3_red_ with Cu(II) by UV–vis spectroscopy (Figure S3). Metal binding or dissociation can be studied by observing the ligand-to-metal charge transfer (LMCT) transition in the middle-to-far UV range.^27,28^ Analysis of the absorption spectrum demonstrates two major LMCT bands centered at 215 and 255 nm, the CysS-Zn(II) LMCT and the CysS-Cu(I) LMCT, respectively. Varying intensities of the LMCT bands result in an isosbestic point at 230 nm (Figure S3). The intensity of the higher energy band (CysS-Zn(II) LMCT) linearly decreases with the addition of successive portions of Cu(II), with a simultaneous linear increase in the intensity of the S–Cu(I) charge transfer band at 255 nm. This trend continues until ∼8 Cu(II) eq are added when the final complex form is formed. This is very consistent with previous spectroscopic results and indicates that Zn_7_MT3_red_ is able to bind and reduce Cu(II) to Cu(I) with concomitant Zn(II) displacement and disulfide formation (Figure S3).^27,28,50^

**Figure 2:**
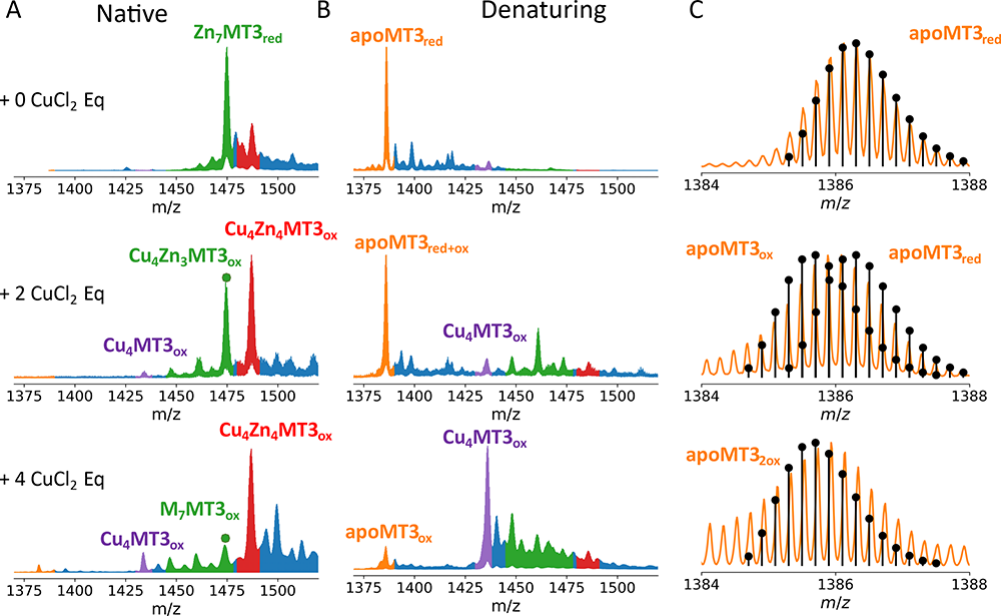
Native mass spectra of Zn_7_MT3 (10 μM) before and after incubation with 2 and 4 CuCl_2_ eq in 200 mM ammonium acetate (pH 6.8) (A). Mass spectra were acquired from (A) after acidification and sprayed under 50:50 H_2_O:ACN and 0.1% formic acid (B). Fitting of the isotopically resolved mass spectrum data to theoretical isotopic distributions (C). Simulations of theoretical isotopic patterns for individual proteins were plotted as stem plots. The molecular formulas can be found in Table S1. The *m*/*z* region corresponds to 5+ ions in all cases. “red” and “ox” subscripts refer to reduced and oxidized (two intramolecular disulfide) MT3 proteins. Note that all complexes of copper are Cu(I), and Zn(II), as discussed in the text.

To confirm the presence of two disulfides in the Cu(I)_4_Zn(II)_4_MT3_ox_ complex, we measured the native and denaturing mass spectra of Zn_7_MT3_red_ and Zn_7_MT3_red_ after the addition of 2 and 4 CuCl_2_ molar eq (Figure 2A,B). We observed that apoMT3 derived from native Zn_7_MT3 could be fit to a reduced state (“red”), and the disulfide formation is a function of CuCl_2_ molar eq (Figure 2C). Upon acidification of the native complex obtained after incubation of Zn_7_MT3_red_ with expected 1:4 stoichiometric CuCl_2_ molar eq, only the oxidized form of apoMT3, apoMT3_ox_, with two disulfides, is visible (Figure 2C). Interestingly, the Cu(I)_4_MT3_ox_ intermediate formed predominates with only a 1:4 Zn_7_MT3:CuCl_2_ stoichiometry (Figure 2B). This shows that the Cu(I)_4_-thiolate cluster is greatly stabilized by the presence of disulfides, likely acting as a steric constraint, preventing the escape of Cu(I) ions. The fitting of the isotopic distributions can be found in Figure S4A,B and Table S1. The disulfides from the Cu(I)_4_Zn(II)_4_MT3_ox_ complex were reduced in the presence of 1 mM TCEP, as observed by the mass shift (Figure S4C).

We then examined the thermodynamics of Zn(II) dissociation in the Cu(I)_4_Zn(II)_4_MT3_ox_ complex by gradually increasing the concentration of EDTA added (Figure S5). We observed a sequential Zn(II) dissociation from the Cu(I)_4_Zn(II)_4_MT3_ox_ complex that leads to the Cu(I)_4_MT3_ox_ intermediate. The Cu(I)-thiolate cluster was then disrupted, resulting in the formation of apoMT3_ox_ with Cu(I)_1_MT3_ox_ as an intermediate. Such a mechanism slightly differs from that in the previous experiment based on cysteine residue protonation (Figure 2). With all this body of work, we may conclude that the oxidized β-domain in full-length MT3 can accommodate up to six Cu(I) ions (Figure 1B), but the product with four Cu(I) seems to be preferred, as it forms a well-defined Cu(I)-thiolate cluster.

### Native and Activated Ion Mobility-Mass Spectrometry to Study Conformational Properties and Stability of Cu(I)/Zn(II)-MT3_ox_ Species

**Figure 3:**
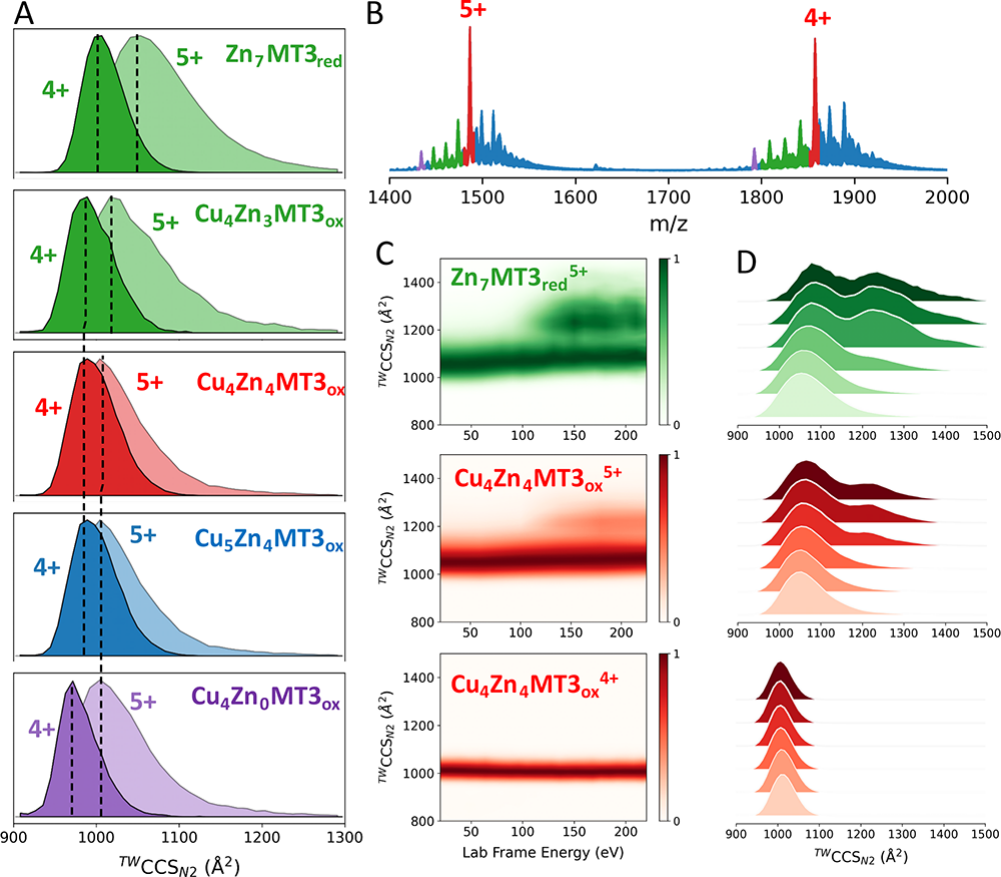
Traveling wave (TW)-derived collision cross section (^*TW*^CCS_*N2*_) profiles (A) and native mass spectra (B) of Zn_7_MT3 upon incubation with 4 CuCl_2_ eq. As a reference, we include the ^*TW*^CCS_*N2*_ profile for Zn_7_MT3 in A. Collision-induced unfolding (CIU) heat maps (C) and ^*TW*^CCS_*N2*_ profiles (D) for the mass-selected Zn_7_MT3_red_^5+^, Cu_4_Zn_4_MT3_ox_^5+^, and Cu_4_Zn_4_MT3_ox_^4+^ ions. The collision cross sections can be found in Table S2.

Following the stoichiometry and thermodynamic characterization of the Cu(II) reaction with Zn_7_MT3_red_, we then attempted to investigate the conformational properties of the Cu(I)_*x*_Zn(II)_*y*_MT3_ox_ complexes by native IM-MS. The estimated ^*TW*^CCS_*N2*_ values are presented in Table S2 and Figure S6. The Zn_7_MT3_red_^5+^ ions exhibit a broad ^*TW*^CCS_*N2*_ distribution, centered at ∼1069 Å^2^, while the ^*TW*^CCS_*N2*_ for Zn_7_MT3_red_^4+^ ions reveal a single compact conformation at 1006 Å^2^ (Figure 3A). The swap from the Zn(II)_3_- to Cu(I)_4_-thiolate cluster induces a structural change that leads to several complexes, including Cu(I)_4_Zn(II)_3_MT3_ox_ species with lower ^*TW*^CCS_*N2*_ values centered at ∼1020 and 992 Å^2^ for 5+ and 4+ ions.

Since the ionic radius of Cu(I) is comparable to that of Zn(II) ions, the presence of two disulfides in the protein together with the Cu(I)_4_-thiolate cluster reduces both its size and flexibility, resulting in narrower ^*TW*^CCS_*N2*_ values. Saturating the protein with one Zn(II), forms the Cu(I)_4_Zn(II)_4_MT3_ox_ species and results in a more compact conformer, with ^*TW*^CCS_*N2*_ values of ∼990 and 980 Å^2^ for 5+ and 4+ ions, respectively. This species represents the maximum number of Zn(II) that can bind in the presence of Cu(I). The addition of one more Cu(II) and formation of the Cu(I)_5_Zn(II)_4_MT3_ox_ species require the protein to open up its structure to accommodate the additional metal ion, as reflected in a slight increase in ^*TW*^CCS_*N2*_ to ∼1020 and 998 Å^2^ for 5+ and 4+ ions, respectively (Figure 3A). On the other hand, one might expect an increase in ^*TW*^CCS_*N2*_ for Cu(I)_4_MT3_ox_ compared to Cu(I)_4_Zn(II)_4_MT3_ox_, as the dissociation of four Zn(II) should result in larger degrees of freedom. However, the cysteines that were bound to Zn(II) formed disulfides, as estimated isotopic pattern fitting, which in turn results in a compact conformation. This is demonstrated by the ^*TW*^CCS_*N2*_ values, which are centered at ∼1000 and 977 Å^2^ for 5+ and 4+ ions, respectively (Table S1). To conclude, looking at the apex of the ^*TW*^CCS_*N2*_ distributions for 4+ ions seems to be useful in accurately discerning structural changes related to the stoichiometry and composition of metal ions and/or disulfides. On the other hand, the ^*TW*^CCS_*N2*_ width for the 5+ ions is more sensitive to metal/disulfide-imposed degrees of freedom.

To examine the stability and dynamics of the Cu(I)_4_Zn(II)_4_MT3_ox_ species formed upon Cu(I)-to-Zn(II) swap and the formation of a Cu(I)_4_-thiolate cluster, we performed IM-MS on mass-selected 5+ and 4+ ions under different collisional activation conditions (Figure 3B–D). At low collision energy (CE), Zn_7_MT3_red_^5+^ ions exhibit a single compact conformation with ^*TW*^CCS_*N2*_ ∼1000 Å^2^, but upon activation, some of the ions shift to extended conformations with ^*TW*^CCS_*N2*_ ∼ 1300 Å^2^. A stable 60:40 ratio of compact to extended conformations was observed across a range of CE. This stability was not due to an oxidation-induced effect that trapped the compact conformer, preventing unfolding (Figure S7). The Cu(I)_4_Zn(II)_4_MT3_ox_^5+^ ions exhibit a more compact and stable conformation than Zn_7_MT3_red_^5+^ ions, with only partial activation upon increasing CE. The Cu(I)_4_Zn(II)_4_MT3_ox_^4+^ ions remained very stable and did not undergo ion activation, supporting the different information provided by the 4+ and 5+ charge states.

### Native Top-Down Mass Spectrometry of Cu(I)/Zn(II)-MT3_ox_ Species

**Figure 4:**
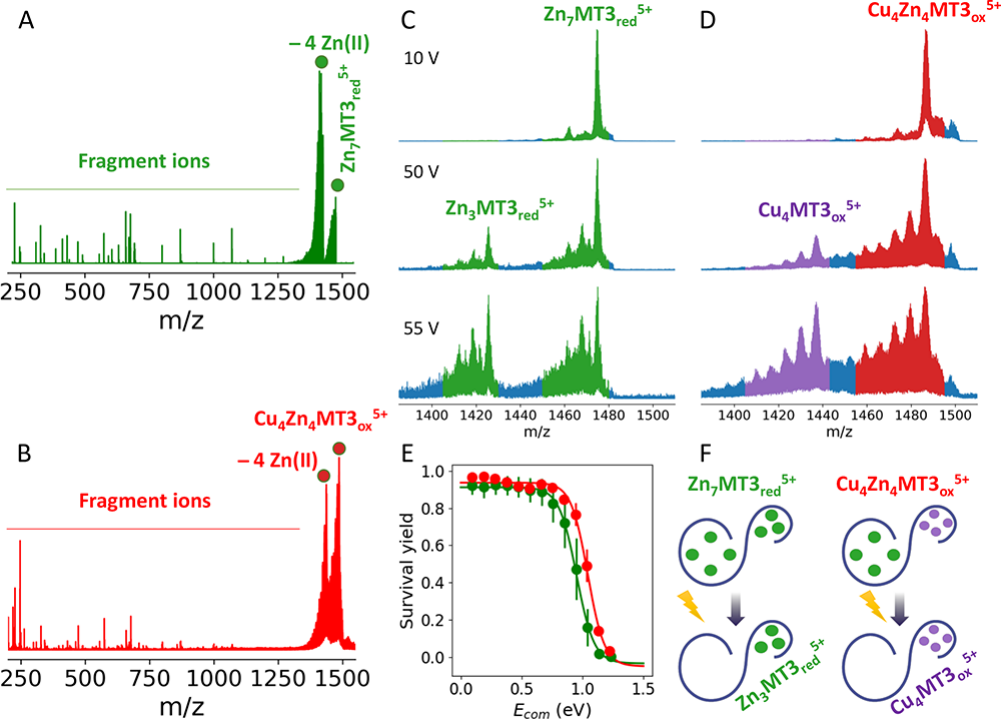
Collision-induced dissociation (CID) experiments. Mass spectra were acquired under different collision energies for quadrupole-selected Zn_7_MT3_red_^5+^ (A,C) and for Cu_4_Zn_4_MT3_ox_^5+^ (B,D). Survival yield plots for quadrupole-selected Zn_7_MT3_red_^5+^ (green) and for Cu_4_Zn_4_MT3_ox_^5+^ (red) (E). Schematic representation of the metal ion dissociation mechanism inferred from the CID experiments (F). The proteins (10 μM) were sprayed in 200 mM ammonium acetate (pH 6.8), and the activation was performed in the trap cell. “red” and “ox” subscripts refer to reduced and oxidized (two intramolecular disulfides) MT3 proteins. Note that all complexes of copper are Cu(I), and Zn(II), as discussed in the text.

A native top-down MS approach was employed to further characterize the metal-protein complexes. The Zn_7_MT3_red_^5+^ and Cu(I)_4_Zn(II)_4_MT3_ox_^5+^ ions were quadrupole-selected and subjected to collision-induced dissociation (CID) (Figure 4A,B). The CID spectra contain two *m*/*z* regions that provide different information. A 250–1250 *m*/*z* region contains fragment ions, and a 1250–1550 *m*/*z* region contains ions corresponding to the precursor and to the precursor with the loss of some metal ions. The latter region reveals interesting clues about the thermodynamic stability of metal-thiolate bonds. In Figure 4C,D, we observed how increasing the collisional activation of both Zn_7_MT3_red_^5+^ and Cu(I)_4_Zn(II)_4_MT3_ox_^5+^ ions yielded the dissociation of four Zn(II) ions, albeit at different activation energies.

To estimate the different relative ion stabilities of the metal-protein complexes, we calculated survival yield (SY) plots of each precursor ion (Figure 4E). SY is defined as the fraction of ions that did not fragment under particular activation energy, here normalized to center-of-mass energies (*E*_com_).^47^ Fitting the data to a sigmoid curve and the derived midpoint, known as *E*_50_, provides a quantitative estimate of the ion stability. In agreement with CIU experiments, the Cu(I)_4_Zn(II)_4_MT3_ox_^5+^ ions were more stable than Zn_7_MT3_red_^5+^ ions (1.04 vs 0.95 eV). We observed a cooperative metal ion dissociation, whereby four metal ions dissociate without the formation of any intermediate upon CID (Figure 4C,D). This mechanism resembles the one obtained when the native metal-protein complex was disrupted by acidification, where the Cu(I)_4_Zn(II)_4_MT3_ox_^5+^ ions yielded Cu(I)_4_MT3_ox_^5+^ (Figure 2). CID promoted dissociation of entropically favored Zn(II) ions, while the enthalpically and entropically favored Cu(I) remained bound to the protein.^40^ Not only does the nature of the metal ion dictate the CID-induced metal dissociation mechanism but also the thermodynamics of each protein domain. Isothermal titration calorimetry (ITC) studies have shown that Zn(II) binding to MT3 is enthalpically disfavored and entropically driven.^40^ The increase in entropy is attributed to Zn(II) and protein desolvation; cysteine deprotonation overcomes the conformational protein entropy induced by metal binding. In light of our results, the four metal ions dissociate from the α-domain, as there is a lower protein conformational entropic penalty than in the β-domain (Figure 4F).

**Figure 5:**
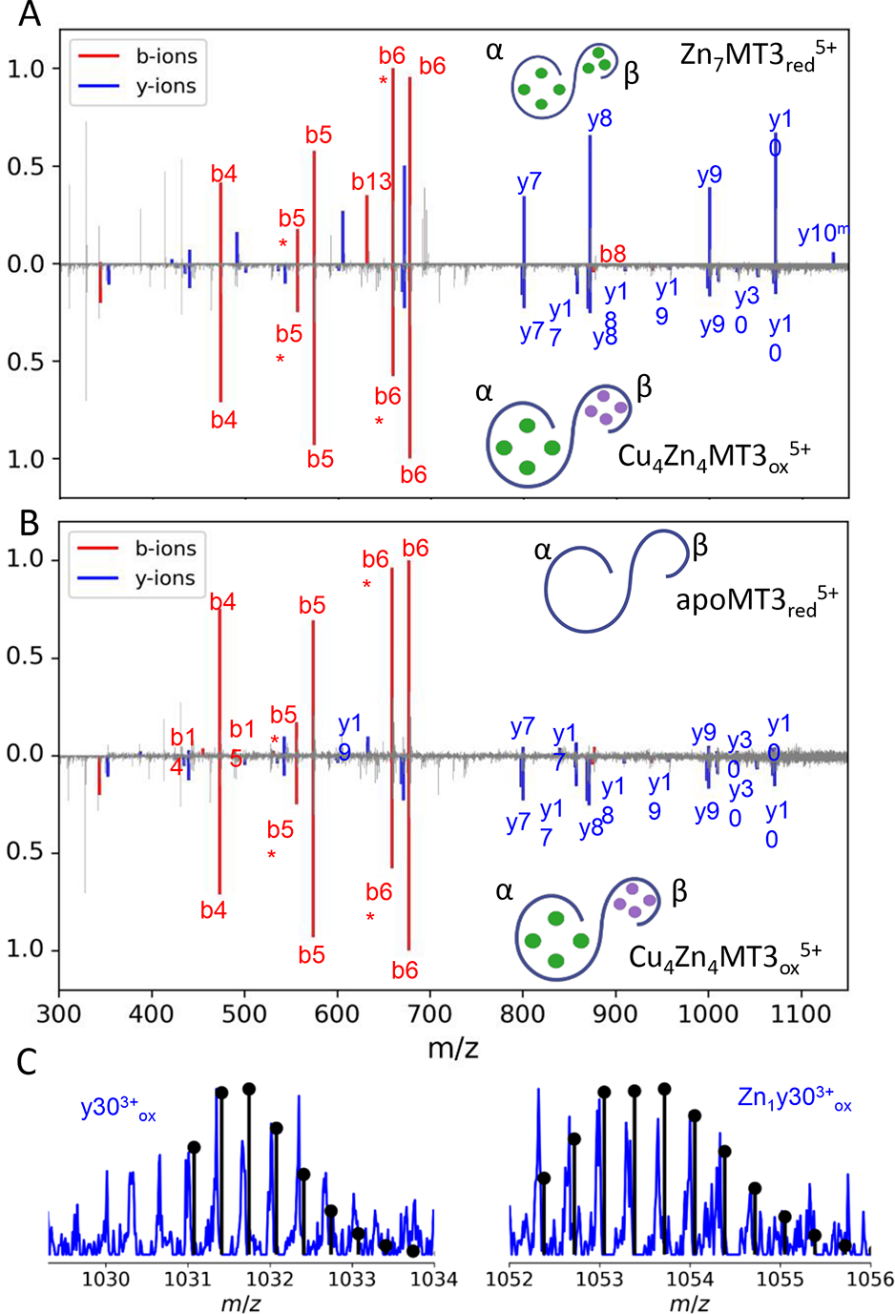
Native top-down CID MS. Mirror fragmentation plot CID acquired for quadrupole-selected Zn_7_MT3_red_^5+^ and Cu_4_Zn_4_MT3_ox_^5+^ (A) and for apoMT3_red_^5+^ and Cu_4_Zn_4_MT3_ox_^5+^ (B). Fitting of the y-fragment ion data to theoretical isotopic distributions (C). Simulations of theoretical isotopic patterns were plotted as stem plots. Identified fragment ions (20 ppm) are colored according to b-ions (red) or y-ions (blue), while the experimental mass spectrum is shown in gray. The asterisk denotes a water loss. All of the fragment ions matched can be found in Tables S3–S5. “red” and “ox” subscripts refer to reduced and oxidized (two intramolecular disulfides) MT3 proteins.

Dissociation of four Zn(II) ions from Zn_7_MT3_red_^5+^ and Cu(I)_4_Zn(II)_4_MT3_ox_^5+^ resulted in Zn_3_MT3_red_^5+^ and Cu(I)_4_MT3_ox_^5+^, and this was followed by covalent bond fragmentation (Figure 4A,B). As a control that the structural changes are imposed by metal binding and not by the protein fold, we performed CID experiments on metal-free apoMT3_red_^5+^. We observed a CID spectrum with extensive metal-free y-fragment ions where the charge is retained by the α-domain C-terminus in both activated Cu(I)_4_Zn(II)_4_MT3_ox_^5+^ and Zn_7_MT3_red_^5+^ ions (Figure 5A). For Zn_7_MT3_red_^5+^, the larger α-domain y-fragment ion corresponds to y10, evidencing that upon Zn(II) dissociation by CID, the remaining three Zn(II) ions are partially redistributed in both the α- and β-domains (Table S4). However, we cannot exclude Zn(II)-induced migration to “non-native” sites as a result of the Zn(II) release by CID. In the case of Cu(I)_4_Zn(II)_4_MT3_ox_^5+^ ions, y-fragment ions appeared, ranging from y2 to y30, clearly supporting our conjecture that the four Cu(I) are bound exclusively to the β-domain (Figure 5A, Table S3). For apoMT3_red_^5+^, y-fragment ions ranging from y7 to y30 were also found (Figure 5B, Table S5). To reinforce our results, we sprayed the Cu(I)_4_Zn(II)_4_MT3_ox_ sample under denaturing conditions where the four Zn(II) dissociated but the Cu(I) remained bound to MT3 forming Cu(I)_4_MT3_ox_. The Cu(I)_4_MT3_ox_^5+^ ions were then mass-selected and subjected to collisional activation (Figure S8A).

We identified characteristic fragment ions and the fragmentation pattern for Cu(I)_4_Zn(II)_4_MT3_ox_^5+^ that clearly confirm our hypothesis. We obtained an almost complete series of y-fragment ions spanning the C-terminal α-domain, except for the range between y20 and y30. Since the α-domain consists of a total of 30 amino acids, our conclusion that the four Cu(I) ions are not bound in the α-domain is reasonable. Otherwise, fragmentation would not be observed because the presence of the metal would provide protection against fragmentation. We rule out the possibility of a Cu(I) ion binding in the 20–30 amino acid region, as a similar fragmentation gap was found for apoMT3, indicating that the absence of fragmentation is not attributed to metal binding but rather to some protein folding effect. We found three characteristic y30 fragment ions that differed in the number of disulfides (Figures 5C, S9). This can be understood as the protein region having a Zn(II) ion bound, and once it dissociates, it is prone to oxidation. As the dissociation of Zn(II) upon ion activation of Cu(I)_4_Zn(II)_4_MT3_ox_^5+^ is not “complete”, we expected to find some Zn(II)-bound fragment ions. Indeed, we observed the same y30 ion carrying a single Zn(II) ion. To further support our findings, we also observed the metal-free y25^3+^ and y30^3+^ ions, but the Zn_1_y30^3+^ disappeared for Cu(I)_4_MT3_ox_^5+^ ions (Figure S8B).

Together, this indicates that the four Zn(II) were bound to the α-domain and the four Cu(I) to the β-domain. In an attempt to further localize the Cu(I) binding sites, we employed top-down electron transfer dissociation (ETD) (Figure S10A). The results showed that no fragmentation occurred via nondissociative electron transfer dissociation (ETnoD),^51^ and additional CID supplementation in the transfer collision cell does not help in obtaining ETD fragmentation (Figure S10B).

Examining the b-type fragment ions corroborated our findings that the α-domain is metal-free and the four Cu(I) are bound in the β-domain. The most abundant β-domain b-fragment ions correspond to b4 to b6. In the absence of metal ions, we observed the most abundant β-domain b-fragment ions correspond to b4 to b6 but also visible are b14 and b15 ions (Figure 5B). Upon Cu(I) binding, b14 and b15 ions disappeared, suggesting that binding of the four Cu(I) takes place in the β-domain protecting toward CID dissociation. Visible ions ranging from b4 to b6 appeared, indicating that the first cysteine residue, Cys6, weakly interacts with Cu(I) ions. For Zn_7_MT3_red_^5+^, we also identified the b13 fragment, stressing that not whole β- or α-domain binds three Zn(II) ions, and these are redistributed in both α- and β-domains (Figure 5A).

## Conclusions

Metallothionein-3 (MT3) is a major player in the regulation of copper and zinc levels in the central nervous system.^52,53^ Unlike other metallothionein isoforms, MT3 is specifically expressed in brain tissue and has distinctive structural characteristics, such as a conserved T_5_CPCP_9_ motif, a Glu23, a Gly24, and an acidic insert in the α-domain (E_55_AAEAE_60_).^6,7,11^ Examining metal-binding properties of engineered MT3 variants showed that the T_5_CPCP_9_ motif and the acidic insert in the α-domain are critical in modulating the Cu(I)/Zn(II) exchange rate.^20^*In vitro* studies have revealed that MT3 has the ability to exchange Zn(II) with Cu(II) that is bound to cellular ligands, such as amyloid β and α-synuclein, which highlights its protective role.^21−25^ An interesting observation is that MT3 can be extracted from mammalian brains in the form of an air-stable complex, Cu(I)_4_Zn(II)_3–4_MT3.^18,19^ A significant body of spectroscopic research has investigated the reaction between Zn_7_MT3 and Cu(II), which has been found to result in the binding and reduction of Cu(II) to Cu(I), dissociation of Zn(II), and formation of two intramolecular disulfides.^27−29,50^ A detailed analysis of the reaction between Zn_7_MT3 with Cu(II) and Cu(I) using electronic absorption and low-temperature luminescence has revealed that the presence of O_2_ does not affect the nature of the products formed and that direct binding of Cu(I) to Zn_7_MT3 does not result in disulfide formation.^28^ Despite the extensive efforts made, it is important to note that the spectroscopic signal obtained represents an average response from the dynamic equilibria between multiple Cu(I)/Zn(II) MT3 species. To gain more precise information about the isolated Cu(I)_4_Zn(II)_3–4_MT3_ox_ complex, this research utilized high-resolution ion-mobility mass spectrometry and various mass spectrometry-based approaches.

Our study utilized native mass spectrometry (MS) to observe the reaction between Zn_7_MT3 and Cu(II) and uncover the resulting interactions. Through fitting isotopically simulated distributions, we were able to assign molecular formulas to each metal-protein complex. Our results show that the β-domain can accommodate up to six Cu(I) ions and form a Cu(I)_6_Zn(II)_4_MT3_ox_ complex. Additionally, we observed the formation of the Cu(I)_4_Zn(II)_4_MT3_ox_ complex as well as partially metal-loaded species, including Cu(I)_4_MT3_ox_ and Cu(I)_4_Zn(II)_1–4_MT3_ox_. Our findings indicate that these species are not a result of gas-phase dissociation but rather the reaction mechanism proceeds through the disassembly of Zn(II) clusters, followed by reassembly into multiple metal-MT3 complexes. The presence of two disulfides in the β-domain of the Cu(I)_4_Zn(II)_4_MT3_ox_ complex was confirmed through mass spectrometry analysis under both native and denaturing conditions and by utilizing the reducing agent TCEP.

We then investigated the thermodynamics of dissociation of Zn(II) ions from the Cu(I)_4_Zn(II)_4_MT3_ox_ complex by gradually increasing the addition of EDTA and through acidification. The metal chelation mechanism occurs in a stepwise sequential manner with EDTA binding to one metal ion at a time, Conversely, acidification demonstrates high cooperativity, leading to the dissociation of all four Zn(II) ions from the Cu(I)_4_Zn(II)_4_MT3_ox_ complex, forming the Cu(I)_4_MT3_ox_ intermediate. The cooperativity arises from the enhancement of proton acceptance by neighboring residues after protonation of one or several cysteine residues, leading to a change in protein conformation. Our findings indicate that, at pH ≈ 4, the Cu(I)_4_Zn(II)_4_MT3_ox_ complex undergoes a series of protonation events, resulting in a stable Cu(I)_4_MT3_ox_ complex state. Such pH corresponds to p*K*_a_′ values of the thiols in high-affinity zinc sites in numerous proteins.^54,55^ This implies that MT3 can serve as an effective copper scavenger under lower pH conditions, as found in certain cellular compartments or physiological states.^56,57^ For instance, *in vitro* studies show that MTs are less susceptible to lysosomal proteolysis when loaded with metals.^58^ The Cu(I)_4_-thiolate cluster is greatly stabilized, possibly due to the disulfides that constrain the Cu(I) ions within the protein. In addition, our metal chelation experiments suggest that upon exposure to cellular apoproteins, the heterogenuous Cu(I)_4_Zn(II)_4_MT3_ox_ species may exchange Zn(II) ions with zinc proteins.^59^

Next, we used native ion mobility-mass spectrometry (IM-MS) to study the conformational characteristics of the Cu(I)_*x*_Zn(II)_*y*_MT3_ox_ complexes observed when Zn_7_MT3_red_ reacts with Cu(II). Our results show that the exchange of three Zn(II) for four Cu(II) in Zn_7_MT3_red_ with subsequent disulfide formation resulting in Cu(I)_4_Zn(II)_4_MT3_ox_ species is accompanied by a reduction in size. This can be attributed to the stronger Cu(I)-thiolate bonds and their more covalent character than in the case of Zn(II)-thiolate as well as the presence of two disulfides in the Cu(I)_4_-thiolate cluster, which both reduce the size and restrict the flexibility of the cluster, resulting in a narrower distribution of traveling wave (TW)-derived collision cross section (^*TW*^CCS_*N2*_) values. Additionally, to gain insight into the stability and dynamics of the formed Cu(I)_*x*_Zn(II)_*y*_MT3_ox_ complexes, we carried out collision-induced unfolding (CIU) experiments. Our results indicate that the Cu(I)_4_Zn(II)_4_MT3_ox_ exhibits a more compact and stable conformation compared to the Zn_7_MT3_red_.

In order to further investigate the stability of the Cu(I)_4_Zn(II)_4_MT3_ox_ complex, we performed collision-induced dissociation (CID) experiments and electron transfer dissociation (ETD). The collisional activation of both Zn_7_MT3_red_^5+^ and Cu(I)_4_Zn(II)_4_MT3_ox_^5+^ ions resulted in the dissociation of four Zn(II) ions without the formation of intermediates. The relative stability of the metal-protein complexes was estimated by calculating survival yield plots, which showed that Cu(I)_4_Zn(II)_4_MT3_ox_^5+^ was more stable than the Zn_7_MT3_red_^5+^ ions. The dissociation of Zn(II) ions is attributed to both the nature of the metal ion and the thermodynamics of the protein domain. Isothermal titration calorimetry studies have shown that Zn(II) binding to MT3 is not enthalpically favored and is entropically driven, and the four metal ions dissociate from the α-domain due to a lower protein conformational entropic penalty than in the β-domain.^40^ The dissociation of Zn(II) ions from Zn_7_MT3_red_^5+^ and Cu(I)_4_Zn(II)_4_MT3_ox_^5+^ ions was followed by covalent bond fragmentation. Experiments on metal-free apoMT3_red_^5+^ showed that the structural changes were due to metal binding rather than the protein fold. The results indicated that while Zn(II) ions are partially distributed in both the α- and β-domains, Cu(I) ions are bound exclusively to the β-domain. The analysis of b-type fragment ions supports the conclusion that the binding of Cu(I) ions in the β-domain protects against CID dissociation. On the other hand, the results for Zn_7_MT3 showed that not all of the β-domain binds Zn(II) ions, and these ions are redistributed in both α- and β-domains.

Thorough using high-resolution mass spectrometry approaches, our study sheds light on the unique function of MT3 among other isoforms by demonstrating the binding of four Zn(II) ions in the α-domain and four Cu(I) ions in the β-domain in the full-length MT3, which not only increases the protein’s metal-binding capacity but also allows it to serve as a Zn(II) delivery system to maintain essential processes while serving as a scavenger for free or protein-bound Cu(II) ions. In the presence of free Cu(II) or Cu(II) donors, Zn(II) ions of Zn_7_MT3 may be exchanged by this metal, protecting the cell from its toxic effects through Cu(II) reduction to Cu(I) and partial β-domain oxidation. Moreover, the stability of this complex at a pH lower than that of cytosolic makes MT3 an ideal acceptor for copper from degraded copper proteins in lysosomes, enabling it to act as a scavenger in cellular compartments with lower pH or in certain pathological states. Despite Zn(II) dissociating easily, Cu(I) remains strongly bound to the protein, providing protection against oxidative damage. Overall, our findings provide a new understanding of the structure, stability, and dynamics of Cu(I)/Zn(II)-MT3, underscoring its crucial role in regulating zinc and copper levels in the human body.

## Supporting Information Available

Materials and methods, expression and purification of metallothionein-3, UV–vis spectroscopy, mass spectrometry and ion mobility, native top-down CID mass spectrometry, Figures S1–S10, Table S1–S6, references (PDF)
