# Supplementary material for: Structural Characterization of Cu(I)/Zn(II)-metallothionein-3 by Ion Mobility Mass Spectrometry and Top-Down Mass Spectrometry
Source: Anal Chem. 2023 Jul 13;95(29):10966–74. doi: 10.1021/acs.analchem.3c00989 (PMC10372872; doi:10.1021/acs.analchem.3c00989)
Supplement: Supplementary file 1 — ac3c00989_si_001.pdf [file ac3c00989_si_001.pdf]

Supporting Information

Structural characterization of Cu(I)/Zn(II) metallothionein-3 by ion mobility mass spectrometry and top-down mass spectrometry

Manuel David Peris-Díaz[ab*], Sylwia Wu[a], Karolina Mosna[a], Ellen Liggett[b], Alexey Barkhanskiy[b], Alicja Orzeł[a], Perdita Barran[b*], Artur Krężel[a*]

[a]*Department of Chemical Biology, Faculty of Biotechnology, University of Wrocław, F. Joliot-Curie 14a, 50-383 Wrocław, Poland* E-mail: manuel.perisdiaz@uwr.edu.pl, artur.krezel@uwr.edu.pl.
[b]*Michael Barber Centre for Collaborative Mass Spectrometry, Manchester Institute of Biotechnology, 131 Princess Street, Manchester, M1 7DN, United Kingdom* E-mail: perdita.barran@manchester.ac.uk.

TABLE OF CONTENTS

Materials. 2
Expression and purification of metallothionein-3 2
UV-vis spectroscopy....3
Mass spectrometry and ion mobility. 3
Native top-down CID mass spectrometry....4
Generation of isotopic patterns, fitting, and scoring....5
Figure S1....6
Figure S2....7
Figure S3....8
Figure S4....9
Figure S5....10
Figure S6....11
Figure S7....12
Figure S8....13
Figure S9....14
Figure S10....15
Table S1 16
Table S2 17

Table S3 18
Table S4 20
Table S5 21
Table S6 22
REFERENCES 24

EXPERIMENTAL SECTION

Materials. The reagents used in this study were purchased from Sigma-Aldrich, Merck, Acros Organics, Roth, BioShop, VWR International (Avantor), and Iris-Biotech GmbH. The following reagents: $ZnSO_4 \cdot 7H_2O$, 4-(2-pyridylazo)resorcinol (PAR), $(NH_4)_2CO_3$, tris(hydroxymethyl)aminomethane (Tris base) and 4-(2-hydroxyethyl)-1 piperazineethanesulfonic acid (HEPES), mass spectrometry grade methanol, tris(2carboxyethyl)phosphine hydrochloride (TCEP), ammonium acetate (AmAc) ethylenediamine-tetraacetic acid (EDTA), and mass spectrometry grade acetonitrile (ACN) were purchased from Sigma-Aldrich. Resin Chelex 100 was acquired from Bio-Rad and 98% hydrochloric acid (HCl) was purchased from VWR Chemicals. Dithiothreitol (DTT) was purchased from Iris Biotech GmbH. Tryptone, LB broth, yeast extract, isopropyl-β-D-1-thiogalactopyranoside (IPTG), and SDS were from Lab Empire, NaCl, NaOH, glycerol, $KH_2PO_4 \cdot H_2O$, K_2HPO_4 from POCH (Gliwice Poland), pTYB21 vector and chitin resin were from New England BioLabs, and 5,5′-dithiobis-(2-nitrobenzoic acid) (DTNB) from TCI Europe N.V. was purchased from Sigma-Aldrich. Ubiquitin (bovine), cytochrome C (equine heart), and β-lactoglobulin (bovine milk) were purchased from Sigma-Aldrich.

Expression and purification of metallothionein-3. Expression vector (Addgene plasmid ID 105710) was transformed into BL21(DE3) *E. coli* cells and growth in culture medium (1.1% tryptone, 2.2% yeast extract, 0.45% glycerol, 1.3% K_2HPO_4, 0.38% KH_2PO_4) at 37°C until ~0.9 OD_{600}. Protein was induced by adding 0.1 mM IPTG supplemented with 0.5 M $ZnSO_4$ to cells and overnight incubation at 20°C with shaking. The next steps of the purification were conducted at 4°C. Cells were collected by centrifugation (4,000 × *g*, 10 min) and resuspended in 50 mL of cold buffer A (20 mM HEPES, pH 8.0, 500 mM NaCl, 1 mM EDTA, 1 mM TCEP), sonicated for 45 min (5 s sonication, 10 s pause cycles) and centrifugation (16,000 × *g*, 15 min). The supernatant was loaded into a chitin resin and incubated overnight with buffer A (20 ml), then washed with buffer A (50 ml) and cleavaged by the addition of 100 mM DTT. The resin was incubated for 48 h at room temperature on a rocking bed. The eluted solution from the

chitin column was concentrated using Amicon Ultra-4 Centrifugal Filter Units with a membrane cut-off of 3 kDa (Merck Millipore, USA) and acidified to pH ~ 2.5 with 7% HCl and subjected to another spin round. The protein was then purified on a size exclusion chromatography HiLoad 16/600 Superdex 75 pg gel filtration column (Cytiva, USA) equilibrated with 10 mM HCl using an AKTA pure system, with a 1 ml/min flow rate.[1] The identity of the eluted protein from SEC was confirmed by ESI-MS using a Bruker Maxis Impact (Bruker Daltonik GmbH, Bremen, Germany) calibrated with a commercial ESI-TOF Tuning mix (Sigma-Aldrich). Thiol concentration was determined spectrophotometrically using a DTNB assay[2], and the Zn(II) binding capacity was confirmed spectrophotometrically by Zn(II) and Cd(II) titrations.[3] To the purified apoMT3, 8.5 molar excess of $ZnSO_4$ was added under a nitrogen blanket, and the pH was adjusted to 8.6 with a 1 M Tris base. Samples were then concentrated with Amicon Ultra-4 Centrifugal Filter Units with a membrane cut-off of *3* kDa (Merck Millipore, USA) and subsequently purified on an SEC HiLoad 16/600 Superdex 75 pg gel filtration column (Cytiva, USA) equilibrated with 20 mM Tris-HCl buffer at pH 8.6. Concentrations of thiols and Zn(II) were determined spectrophotometrically using DTNB and PAR assays, respectively.[4]

UV-vis spectroscopy. The spectroscopic titrations were performed in anaerobic conditions, and all reagents were degassed before being placed in the glove box. The reaction of 25 μM Zn_7MT3_{red} with Cu(II) ($CuCl_2$ in 20 μM HCl) in chelexed borate buffer (100 mM, NaF 100 mM, pH 7.4) was monitored in the wavelengths of 210-450 nm. The samples were equilibrated over 2.5 min. This time was sufficient to reach a plateau in the kinetic mode at 255 nm.

Mass spectrometry and ion mobility. MS and IM-MS experiments were carried out on a Synapt XS HDMS equipped with nanoelectrospray ionization (Waters Corporation, Manchester, UK). Samples were prepared at 10-20 μM in 200 mM ammonium acetate (AmAc), pH 6.8 and when indicated supplemented with 1 mM TCEP, and desalted using micro Bio-Spin 6 columns (Bio-Rad). 3-10 μL of sample were loaded into borosilicate glass capillaries (O.D. 1.2 mm, I.D. 0.9 mm, World Precision Instruments, Stevenage, UK) produced in-house using a Flaming/Brown P-1000 micropipette puller (Sutter Instrument Co., Novato, CA, USA) and ions were produced by applying a positive potential of 0.9-1.4 kV via a platinum wire (Goodfellow). All of the experiments were performed in sensitivity mode to maximize ion transmission. Native IM-MS experiments were done by using source conditions (source temperature 30°C, cone voltage 20 V, source offset 1), trap (5 V) collision energy as well as bias potentials (trap DC bias 35 V) that prevented ion activation. The helium cell and nitrogen

IM gases were operated at 200 and 75 mL·min^{-1}, respectively. Two sets of TWIMS parameters of travelling wave (TW) velocity and height were used, 300 ms^{-1} and 20 V, and 480 ms^{-1} and 20 V. Collision-induced unfolding (CIU) experiments were performed by increasing trap collision energies (0-60 V range) of quadrupole-selected ions and recording ion arrival time distributions. Ion activation energies were reported as laboratory frame energy (E_{lab}) to account for the charge state of the quadrupole-selected ion. The time of flight was calibrated using 2 µg·µL^{-1} NaI made up in 1:1 water:isopropanol and the arrival time distributions were calibrated to $^{TW}CCS_{N2}$ using a TWIMS calibration procedure. We used ubiquitin (bovine), cytochrome C (equine heart), and β-lactoglobulin (bovine milk) to calibrate the TW device. The lyophilized powders were dissolved in either 200 mM AmAc or in 50:50 H_2O:acetonitrile (ACN) and 0.1% formic acid and diluted to a 10 µM protein concentration. We calculated TW-derived CCS under native or denaturing calibrant conditions, and with two different T-Wave velocities (300 and 480 ms^{-1}). IM-MS data was recorded on three different days. The literature CCS_{N2} values for the standards were obtained from A. P. France *et al.*[5] Data were analysed by means MassLynx v4.2 (Waters Corp., UK), ORIGAMI[6] and custom scripts in Python 3.5 (available in https://github.com/ManuelPerisDiaz/Cu-Zn-MT3).

Native top-down CID mass spectrometry. Native top-down collision-induced dissociation (CID) mass spectrometry experiments were performed by applying 20-60 V of trap collision energies of quadrupole-selected ions with argon as the collision gas. Survival yield curves of precursor ions were calculated according to eq 1:

$$\mathrm{SY} = \frac{I_{\mathrm{P}}}{I_{\mathrm{P}} + \Sigma I_{\mathrm{F}}} \qquad \text{(S1)}$$

where I_P and I_F refers to the intensity of precursor and fragment ions, respectively.[7] Trap voltages ($trap_{CE}$) were transformed to center-of-mass energies (E_{com}) using eq 2:

$$E_{\mathrm{com}} = \frac{\mathrm{m_g}}{\mathrm{m_g} + \mathrm{m_p}} \cdot \mathrm{trap_{CE}} \cdot z \qquad \text{(S2)}$$

where m_g, m_p and z denote the mass gas (N_2) and mass and charge state of precursor ion, respectively. The survival plots (SY vs Ecom) were fitted to a sigmoid function using non-linear least squares implemented in SciPy 1.10.0.[8] Averaged raw mass spectrum were converted to txt file format, and mMass was used for peak picking (S/N threshold 3) and deisotoping (isotope mass tolerance 0.05 m/z and isotope intensity tolerance 50%).[9] Experimental peak list was matched against a theoretical list of b/y fragment ions including neutral losses within a 20

ppm accuracy threshold. The results were exported as csv files for further analysis with custom Python 3.5 scripts.

Generation of isotopic patterns, fitting, and scoring. To accurately determine the stoichiometry of metal-protein complexes and assign fragment ions to experimental data, multiple theoretical protein/peptide isotopic distributions were generated, and the one with the best fit was selected. The Python 3.5 script can be found in https://github.com/ManuelPerisDiaz/Cu-Zn-MT3). The script works as follows:

1. Generation of molecule candidates. In the first step, the *generate_molecules* function takes three ranges (h_range, zn_range, cu_range) as input parameters. It generates a list of molecules based on these ranges and fixed elemental compositions. The fixed element, namely 'C', 'N', 'O', and 'S', along with their corresponding counts are utilized. The function iterates over the ranges of hydrogen (H), zinc (Zn), and copper (Cu) using nested for loops. For each combination of H, Zn, and Cu values, a new molecule composition is created by copying the fixed elements and updating the counts of H, Zn, and Cu. The resulting composition is then appended to the list of molecules. Finally, the function returns the generated list of molecules.
2. Peak detection: The experimental data is loaded, and the *find_peaks* function from scipy.signal is employed to find peaks in the experimental data. Peak detection is based on a minimum peak height of 20 %.
3. Iteration over molecules: For each molecule in the list of molecule candidates, a loop generates a theoretical isotopic pattern, then it is filtered based on intensity threshold (20% by default). The filtered isotopic pattern is interpolated onto the m/z values of the experimental data.
4. The R2 and chi-square metrics is calculated for each one of them. After iterating over all molecules, the loop outputs the value and the best molecule found based on the chi-square metric, and the R2 metric. Essentially, the loop determines the molecule with the best R2 metric and the molecule with the best chi-square metric among all the molecules in the list.
5. The script generates a csv file that includes the formula, R2, and chi-square values for each molecule, and a plot for each candidate molecule.

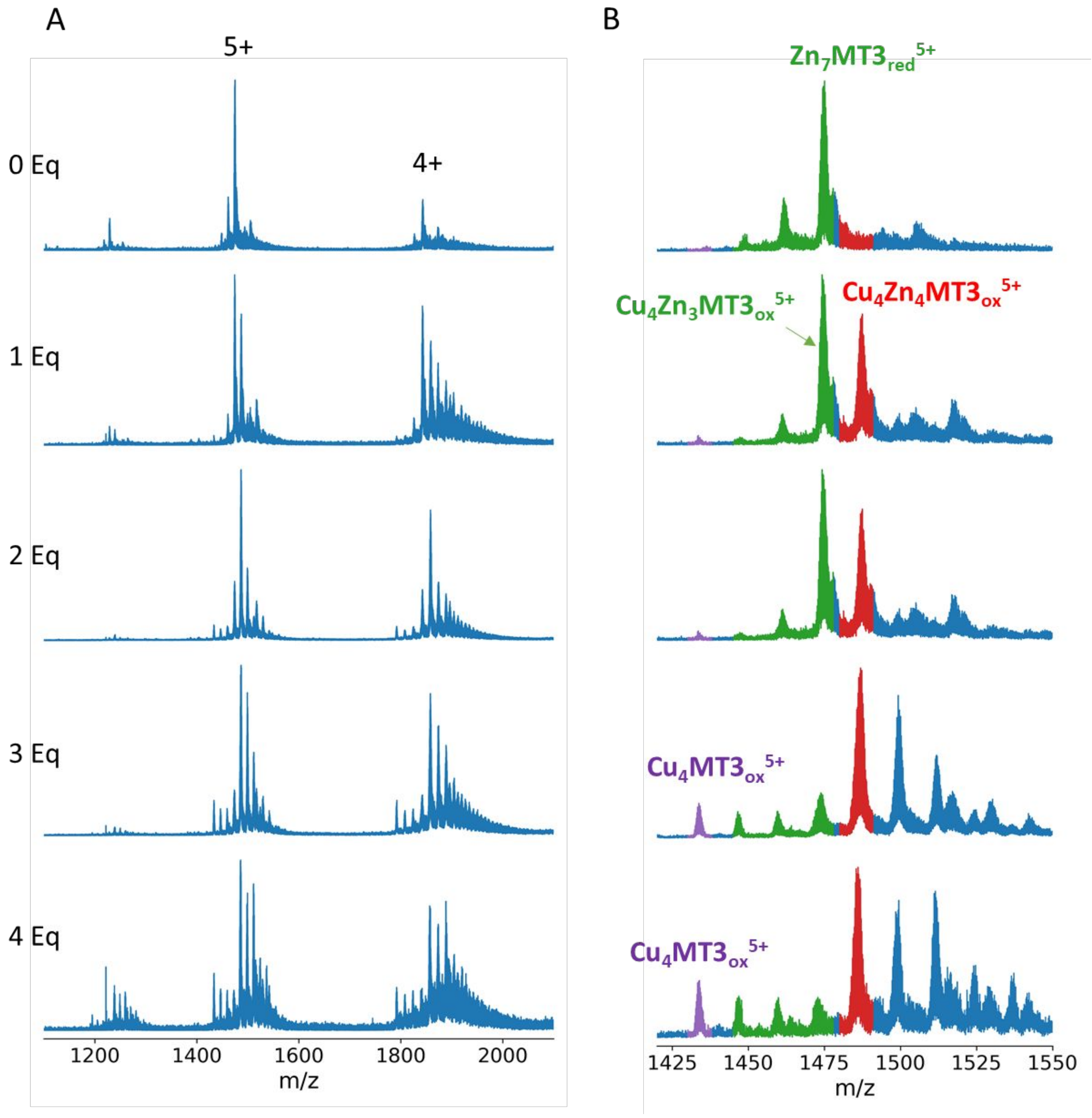

Figure S1. Native mass spectra of Zn_7MT3_{red}, and the products upon addition of 1, 2, 3 and 4 $CuCl_2$ equivalents (A). The *m/z* region correspond to 5+ ions is shown in (B). The proteins (10 μM) were sprayed in 200 mM ammonium acetate (pH 6.8). "red" and "ox" subscripts refer to reduced and oxidized (2 intramolecular disulfides) MT3 proteins. Note that all complexes of copper are Cu(I), and Zn(II), as discussed in the text.

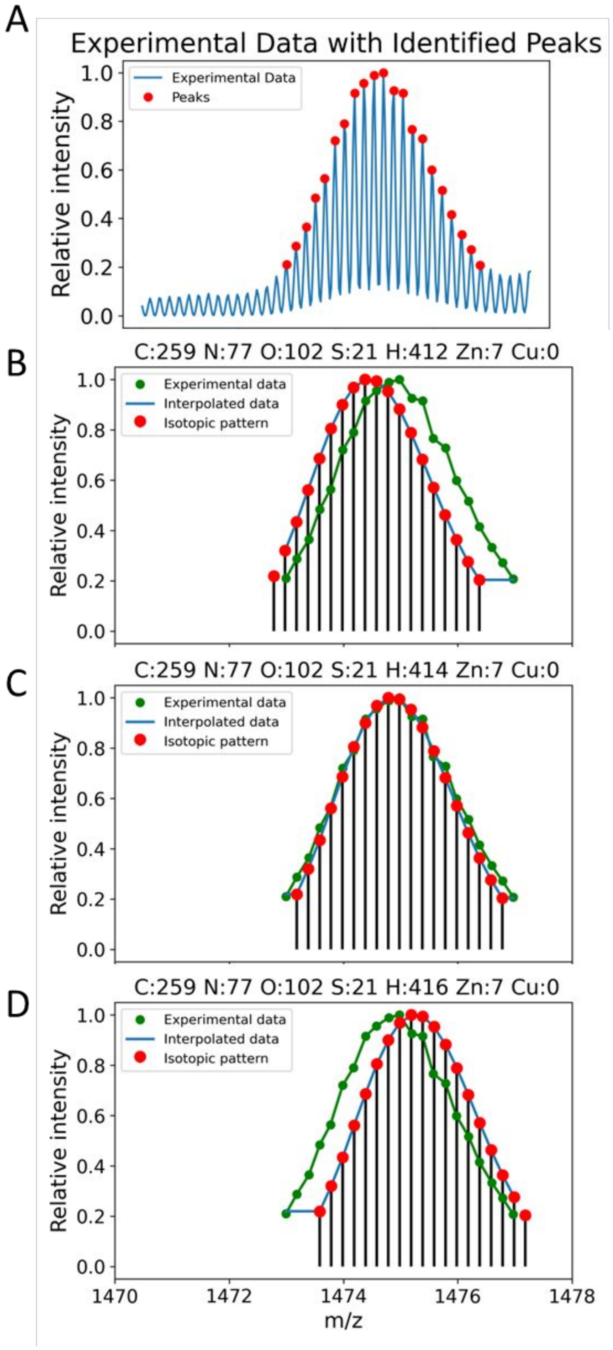

Figure S2. Computational workflow for generating multiple isotopic patterns, fitting to the experimental data and scoring the results. Experimental data with the detected peaks for $Zn_7MT3_{red}^{5+}$ (A). Simulations of theoretical isotopic patterns for $Zn_7MT3_{red}^{5+}$ with variable proton number ("H") were plotted as stem plots (B-D). The filtered theoretical isotopic pattern was filtered based on intensity threshold (20%), and then interpolated onto the m/z values of the experimental data (blue line). The script indicated that $C_{259}H_{414}O_{102}N_{77}S_{21}Zn_7$ provided the best R2 (0.98) and the lowest chi-square (0.0916) among all the candidates assayed.

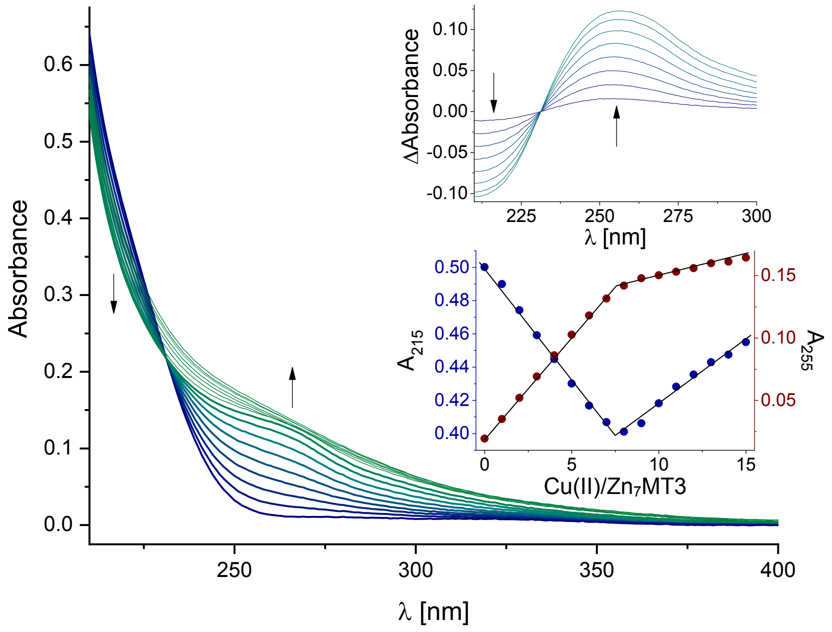

Figure S3. Absorption spectra obtained upon reaction of 25 μM Zn_7MT3 in 100 mM borate buffer (100mM NaF, pH 7.4) with 0–15 eq of $CuCl_2$. Upper inset: the differential absorption spectra at wavelengths of 210-300 nm obtained by subtracting the Zn_7MT3 spectrum from each spectrum after adding $CuCl_2$, in the range of 1–8 $CuCl_2$ Eq. Bottom inset: changes at 215 nm and 255 nm caused by the addition of 0–15 $CuCl_2$ Eq.

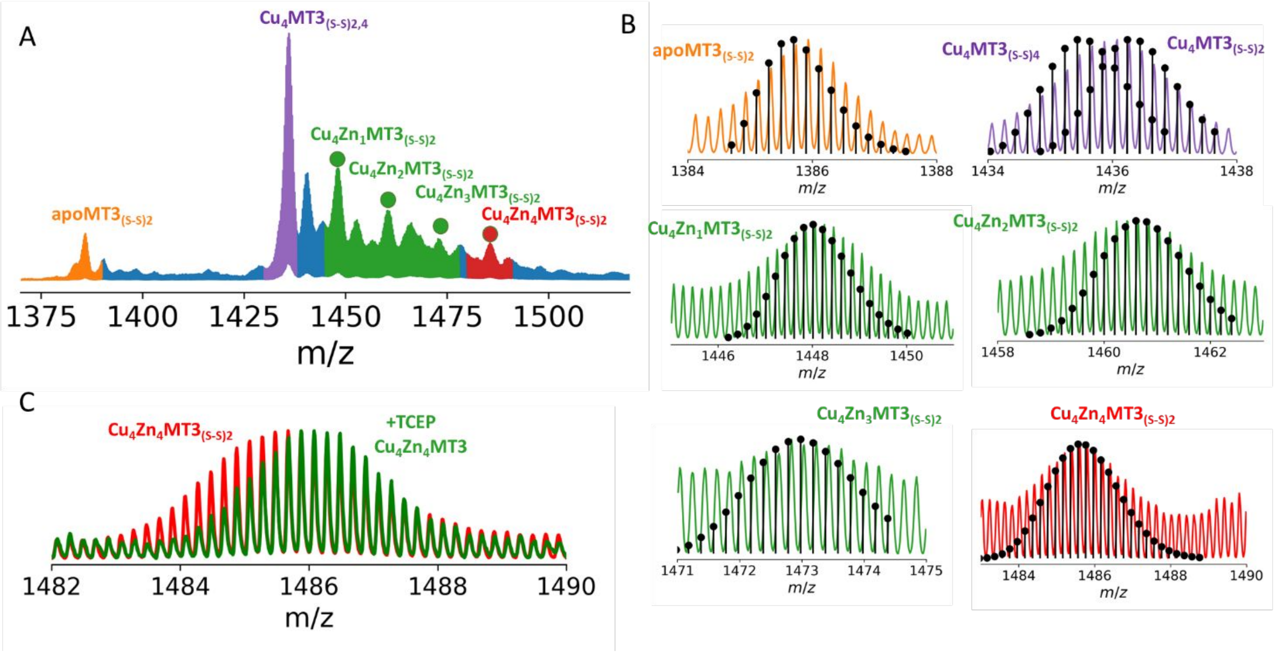

Figure S4. Native mass spectra of Zn_7MT3 incubated with 4 $CuCl_2$ (A). The *m/z* region correspond to 5+ ions. Fitting of the isotopically-resolved mass spectrum data to theoretical isotopic distributions (B). Simulations of theoretical isotopic patterns for individual proteins were plotted as stem plots. The molecular formulas can be found in Table S1.

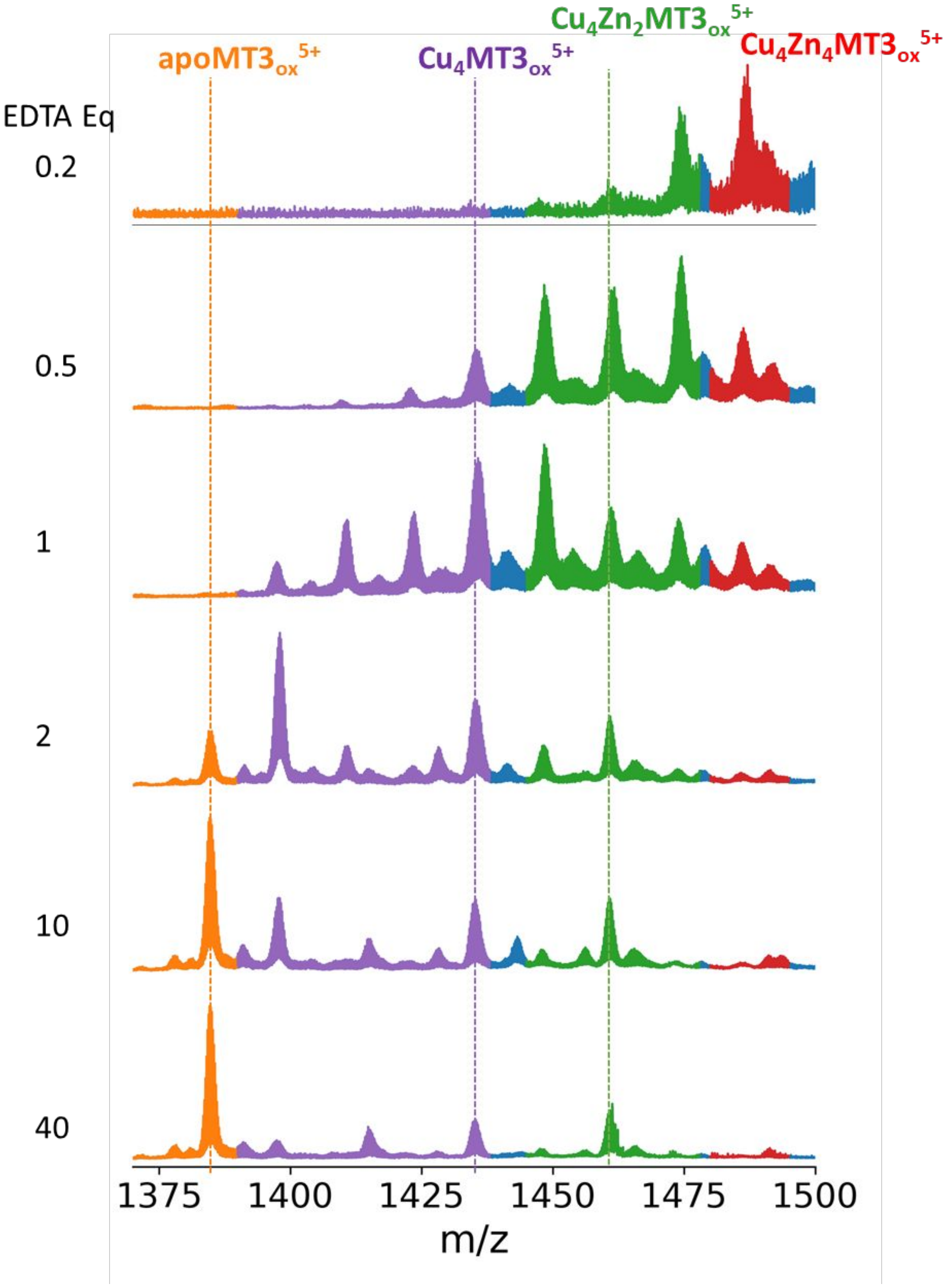

Figure S5. Native mass spectra of Zn_7MT3 incubated with increasing Eq of metal chelator EDTA. The m/z region correspond to 5+ ions. "ox" subscript refer to oxidized (2 intramolecular disulfides) MT3 proteins. Note that all complexes copper is Cu(I), and Zn(II), as discussed in the text.

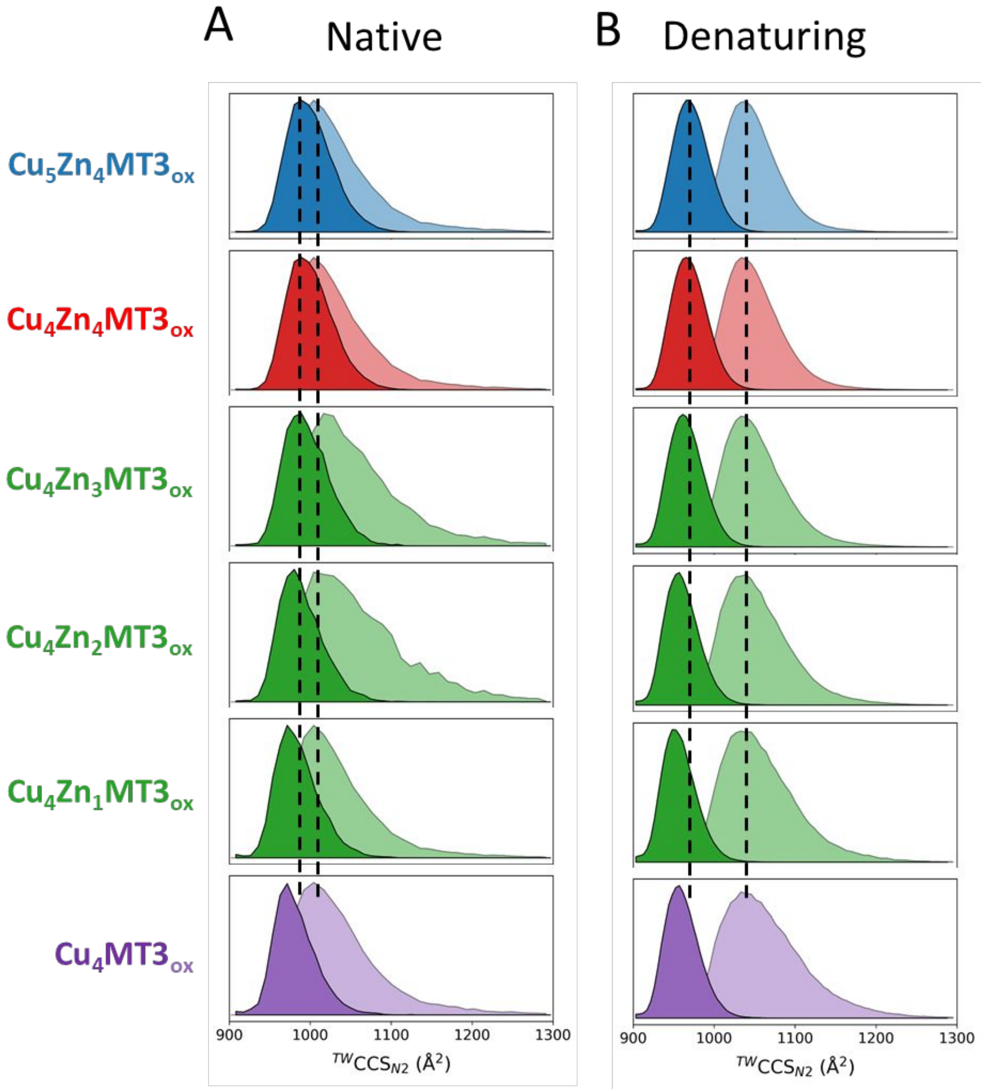

Figure S6. Collision cross sections (CCS) profiles of the products found upon incubation of Zn_7MT3 with 4 $CuCl_2$ equivalents calculated using native (A) or denaturing IM calibration (B). The darker coloured CCS distributions (left ones within the same plot) corresponds to the 4+ ions, while the smoothed ones (right ones) corresponds to the 5+ ions, as shown in Figure 3. The proteins (10 µM) were sprayed in 200 mM ammonium acetate (pH 6.8). The collision cross sections can be found in Table S2. "ox" subscript refer to oxidized (2 intramolecular disulfides) MT3 proteins. Note that all complexes of copper are Cu(I), and Zn(II), as discussed in the text.

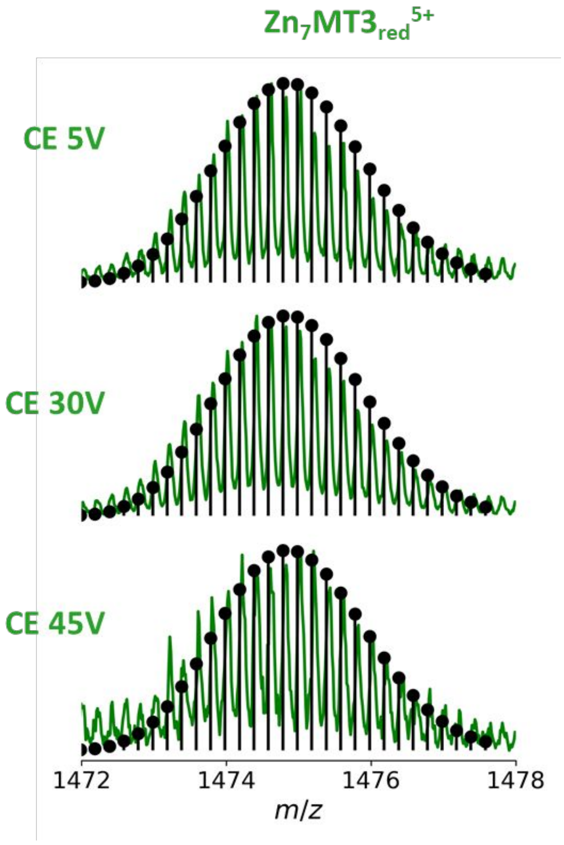

Figure S7. Signals corresponding to mass-selected $Zn_7MT3_{red}^{5+}$ ions at different collision energies used in the collision-induced unfolding (CIU) experiments shown in Figure 3. The proteins (10 µM) were sprayed in 200 mM ammonium acetate (pH 6.8). “red” subscripts refer to reduced MT3 proteins.

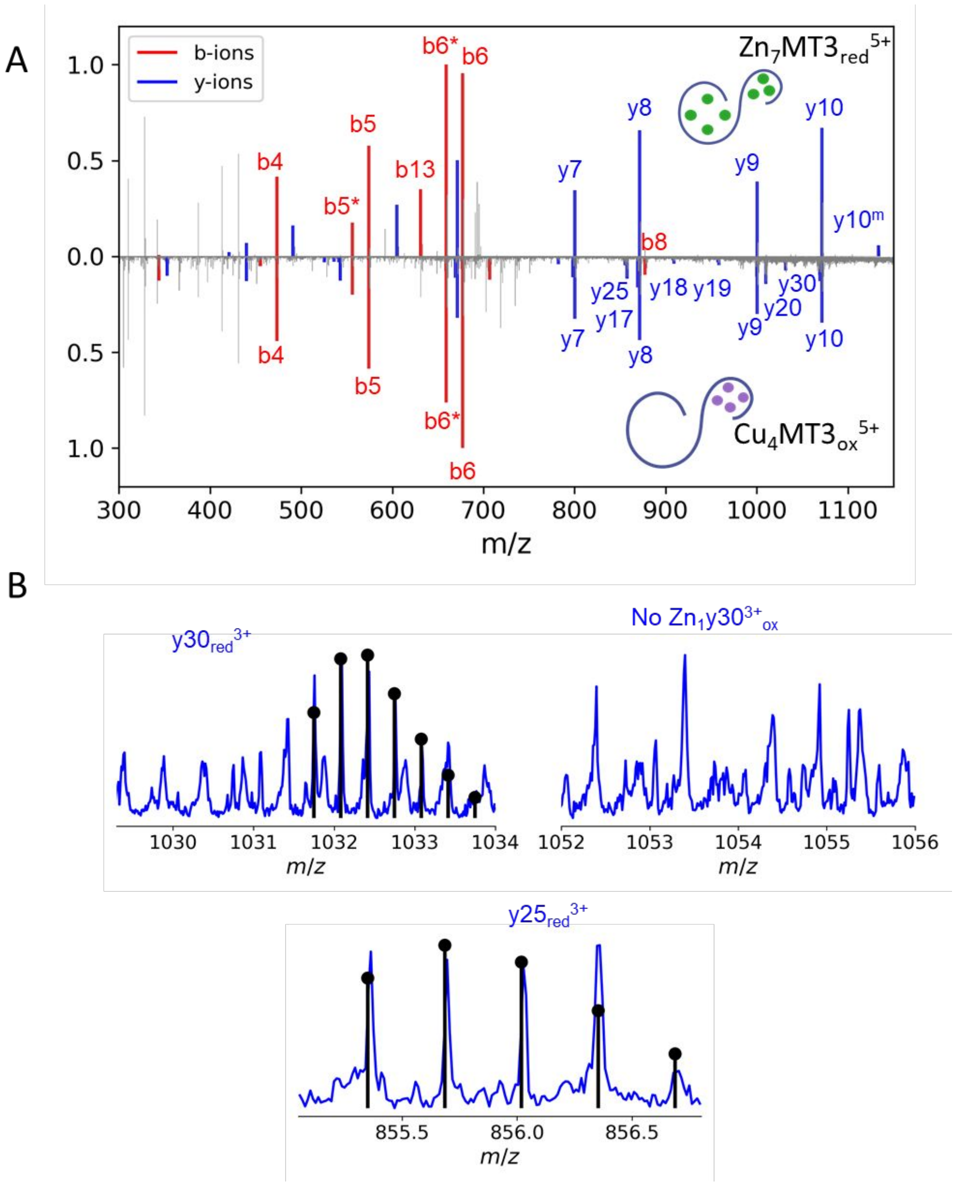

Figure S8. Top-down CID MS for mass-selected $Cu(I)_4MT3_{ox}^{5+}$ ions (A). Fitting of the y-fragment ions data to theoretical isotopic distribu-tions (B). The proteins (5 μM) were sprayed in denaturing conditions. “red” and “ox” subscripts refer to reduced and oxidized (2 intramolecular disulfides) MT3 proteins. Note that all complexes of copper are Cu(I), and Zn(II), as discussed in the text.

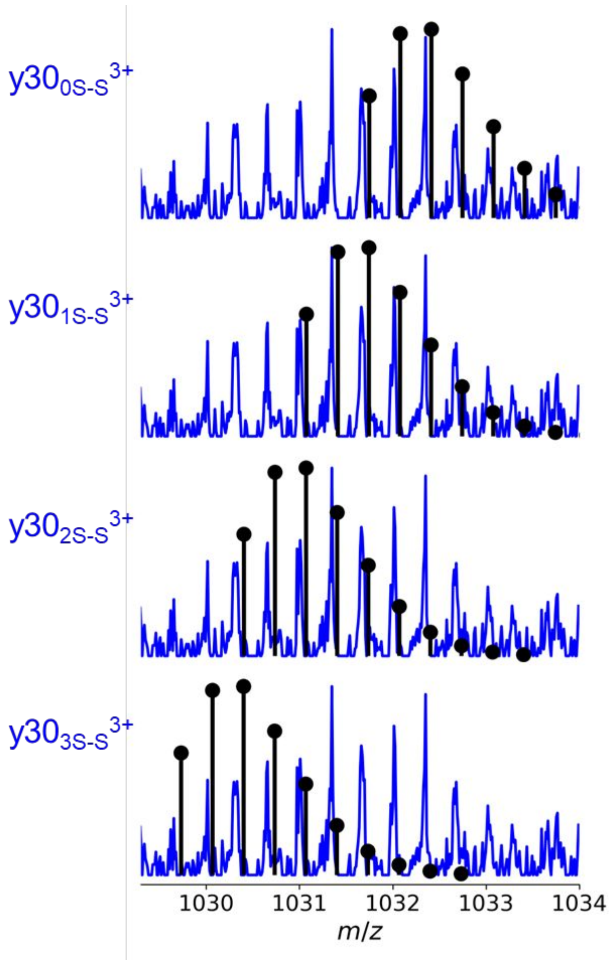

Figure S9. Signals corresponding to $y30^{3+}$ ions with different disulfide bonds (S-S) were obtained from a top-down CID MS experiment for mass-selected $Cu(I)_4Zn(II)_4MT3_{ox}{}^{5+}$ ions. Theoretical simulations of isotopic patterns were plotted as stem plots, while the experimental mass spectrum is shown in blue.

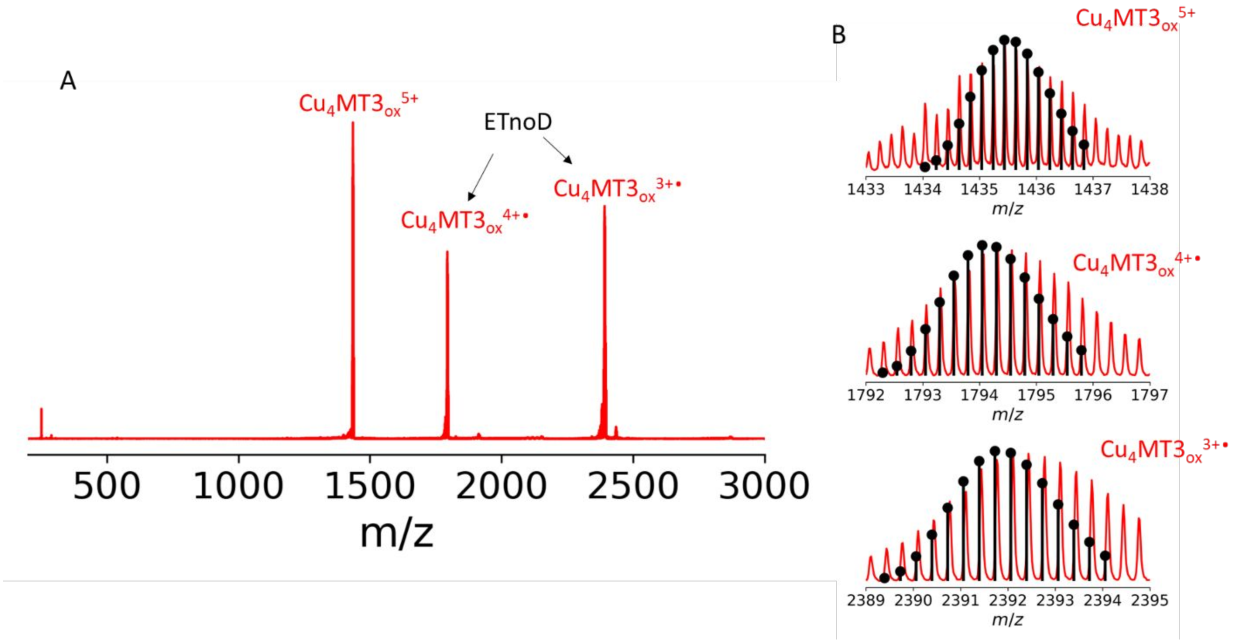

Figure S10. Top-down electron transfer dissociation (ETD) MS for mass-selected Cu(I)$_4$MT3$_{ox}^{5+}$ ions. The proteins (5 µM) were sprayed in denaturing conditions. "ox" subscript refer to oxidized (2 intramolecular disulfides) MT3 proteins. Note that all complexes of copper are Cu(I) as discussed in the text.

Table S1. Accurate mass measurements of Cu(I)/Zn(II) metallothionein-3 complexes by native MS. MAE refers to the mean absolute mass error calculated as the mean error between the experimental and the fitted molecular formula.

Protein	formula	MAE (Da)	R^2
Zn_7MT3_{red}	$C_{259}H_{414}O_{102}N_{77}S_{21}Zn_7$	0.017	0.98
$Cu(I)_6Zn(II)_4MT3_{ox}$	$C_{259}H_{413}O_{102}N_{77}S_{21}Zn_4Cu_6$	0.018	0.93
$Cu(I)_5Zn(II)_4MT3_{ox}$	$C_{259}H_{414}O_{102}N_{77}S_{21}Zn_4Cu_5$	0.018	0.82
$Cu(I)_4Zn(II)_4MT3_{ox}$	$C_{259}H_{414}O_{102}N_{77}S_{21}Zn_4Cu_4$	0.019	0.95
$Cu(I)_4Zn(II)_3MT3_{ox}$	$C_{259}H_{416}O_{102}N_{77}S_{21}Zn_3Cu_4$	0.021	0.80
$Cu(I)_4Zn(II)_2MT3_{ox}$	$C_{259}H_{416}O_{102}N_{77}S_{21}Zn_2Cu_4$	0.018	0.65
$Cu(I)_4Zn(II)_1MT3_{ox}$	$C_{259}H_{415}O_{102}N_{77}S_{21}Zn_1Cu_4$	0.018	0.64
$Cu(I)_4MT3_{ox}$	$C_{259}H_{414}O_{102}N_{77}S_{21}Cu_4$	0.014	0.70

Table S2. Collision cross sections (CCS) of 10 μM $Zn_xCu_yMT3_{ox}$ and Zn_7MT3_{red} in 200 mM ammonium acetate. "n" and "a" refers to two different conditions used to calibrate the TW device. In "n", the calibrants were dissolved in 200 mM ammonium acetate, and the TW set up to 300 m/s and 20 V IMS wave velocity and wave height, respectively. In "a" calibrants were dissolved in 50:50 ACN:H2O 0.1 % FA, and the TW set up to 480 m/s and 20 V IMS wave velocity and wave height, respectively.

Complex	*z*	*m/z*	$^{TW}CCS_{N2}$ (Å^2) (mean ± sd)
$Cu(I)_5Zn(II)_4MT3_{ox}$	$[M+4H]^{4+}$	1873	970 ± 5[a]<br>998 ± 2[n]
	$[M+5H]^{5+}$	1499	1041 ± 6[a]<br>1020 ± 7[n]
$Cu(I)_4Zn(II)_4MT3_{ox}$	$[M+4H]^{4+}$	1857	968 ± 4[a]<br>980 ± 2[n]
	$[M+5H]^{5+}$	1486	1042 ± 6[a]<br>988 ± 8[n]
$Cu(I)_4Zn(II)_3MT3_{ox}$	$[M+4H]^{4+}$	1842	963 ± 4[a]<br>992 ± 2[n]
	$[M+5H]^{5+}$	1474	1043 ± 7[a]<br>1020 ± 14[n]
$Cu(I)_4Zn(II)_2MT3_{ox}$	$[M+4H]^{4+}$	1825	958 ± 4[a]<br>984 ± 3[n]
	$[M+5H]^{5+}$	1460	1044 ± 8[a]<br>1020 ± 19[n]
$Cu(I)_4Zn(II)_1MT3_{ox}$	$[M+4H]^{4+}$	1809	954 ± 4[a]<br>980 ± 3[n]
	$[M+5H]^{5+}$	1446	1046 ± 10[a]<br>1010 ± 7[n]
$Cu(I)_4MT3_{ox}$	$[M+4H]^{4+}$	1793	958 ± 4[a]<br>977 ± 3[n]
	$[M+5H]^{5+}$	1434	1050 ± 11[a]<br>1004 ± 7[n]
Zn_7MT3_{red}	$[M+4H]^{4+}$	1843	962 ± 1[a]<br>1006 ± 1[n]
	$[M+5H]^{5+}$	1474	1052 ± 2[a]<br>1069 ± 5[n]

Table S3. List of fragment ions identified for the CID spectrum of quadrupole-selected $Cu(I)_4Zn(II)_4MT3_{ox}^{5+}$.

Ion	Slice	*m/z*	*z*	Sequence	Error (ppm)
b2 -H_2O	[1-2]	229.0641	1	.MD.p	-0.5
y2 -NH_3	[67-68]	232.0512	1	c.CQ. [1xCystine]	-2.4
b2	[1-2]	247.0747	1	.MD.p	17.6
y2	[67-68]	249.0778	1	c.CQ. [1xCystine]	-18.1
y2	[67-68]	250.0856	1	c.CQ.	16.9
b3	[1-3]	344.1275	1	.MDP.e	2.6
y3	[66-68]	353.0948	1	s.CCQ.	10.3
y8 -H_2O	[61-68]	426.6498	2	e.AEKCSCCQ. [1xCystine]	2.7
y8	[61-68]	435.1511	2	e.AEKCSCCQ. [2xCystine]	16.9
y4	[65-68]	440.1268	1	c.SCCQ.	0.9
b4	[1-4]	473.1701	1	.MDPE.t	-1.9
y9	[60-68]	500.6803	2	a.EAEKCSCCQ.	4.3
y10	[59-68]	535.1910	2	e.AEAEKCSCCQ. [2xCystine]	0.3
y5	[64-68]	543.1360	1	k.CSCCQ.	-14.9
b5 -H_2O	[1-5]	556.2072	1	.MDPET.c	3.5
b5	[1-5]	574.2177	1	.MDPET.c	-2.9
y11	[58-68]	599.7123	2	a.EAEAEKCSCCQ. [2xCystine]	-12.4
y11	[58-68]	600.2162	2	a.EAEAEKCSCCQ. [1xCystine]	-4.9
y11	[58-68]	600.7201	2	a.EAEAEKCSCCQ.	-0.5
b6 -H_2O	[1-6]	658.2085	1	.MDPETC.p [1xCystine]	-5
b6 -H_2O	[1-6]	659.2164	1	.MDPETC.p	0.1
y6	[63-68]	669.2153	1	e.KCSCCQ. [2xCystine]	-1.7
y6	[63-68]	671.2310	1	e.KCSCCQ.	-3.5
b6	[1-6]	677.2269	1	.MDPETC.p	-4.9
y7	[62-68]	798.2579	1	a.EKCSCCQ. [2xCystine]	-2.3
y7	[62-68]	800.2736	1	a.EKCSCCQ.	-1.7
y17	[52-68]	856.3397	2	c.KGGEAAEAEAEKCSCCQ. [2xCystine]	-2.4
y17	[52-68]	856.8436	2	c.KGGEAAEAEAEKCSCCQ. [1xCystine]	2.2
y17	[52-68]	857.3475	2	c.KGGEAAEAEAEKCSCCQ.	3.5
y8	[61-68]	869.2950	1	e.AEKCSCCQ. [2xCystine]	4.5
y8	[61-68]	871.3107	1	e.AEKCSCCQ.	0.8
b8	[1-8]	875.2732	1	.MDPETCPC.p [2xCystine]	-10.1
y18	[51-68]	908.8521	2	v.CKGGEAAEAEAEKCSCCQ.	-12.3
y19	[50-68]	957.8824	2	c.VCKGGEAAEAEAEKCSCCQ. [1xCystine]	-1.9
y9	[60-68]	998.3376	1	a.EAEKCSCCQ. [2xCystine]	6
y9	[60-68]	1000.3533	1	a.EAEKCSCCQ.	-10.5
y20	[49-68]	1008.8830	2	d.CVCKGGEAAEAEAEKCSCCQ. [2xCystine]	5
y20	[49-68]	1009.8909	2	d.CVCKGGEAAEAEAEKCSCCQ.	-2.5

y30	[39-68]	1030.0658	3	c.PAECEKCAKDCVCKGGEAAEAEAEKCSCCQ. [5xCystine]	16.8
y30	[39-68]	1030.7377	3	c.PAECEKCAKDCVCKGGEAAEAEAEKCSCCQ. [3xCystine]	-13.4
y30	[39-68]	1031.0736	3	c.PAECEKCAKDCVCKGGEAAEAEAEKCSCCQ. [2xCystine]	7.6
y30	[39-68]	1051.7114	3	c.PAECEKCAKDCVCKGGEAAEAEAEKCSCCQ. [4xCystine; 1xZn]	9
y30	[39-68]	1052.3833	3	c.PAECEKCAKDCVCKGGEAAEAEAEKCSCCQ. [2xCystine; 1xZn]	12
y30	[39-68]	1053.0552	3	c.PAECEKCAKDCVCKGGEAAEAEAEKCSCCQ. [1xZn]	13.2
y10	[59-68]	1069.3747	1	e.AEAEKCSCCQ. [2xCystine]	7.9
y10	[59-68]	1070.3826	1	e.AEAEKCSCCQ. [1xCystine]	12.6
y10	[59-68]	1071.3904	1	e.AEAEKCSCCQ.	3.2

Table S4. List of fragment ions identified for the CID spectrum of quadrupole-selected $Zn_7MT3_{red}^{5+}$.

Ion	Slice	*mz*	*z*	Sequence	Error (ppm)
b2	[1-2]	247.0747	1	.MD.p	1.2
y2	[67-68]	250.0856	1	c.CQ.	1.2
b3	[1-3]	344.1275	1	.MDP.e	0.1
y10	[59-68]	421.0589	3	e.AEAEKCSCCQ. [2xCystine; 1xZn3]	-16.5
y4	[65-68]	440.1268	1	c.SCCQ.	-2.0
b4	[1-4]	473.1701	1	.MDPE.t	-0.3
y8 -NH_3	[61-68]	491.0709	2	e.AEKCSCCQ. [1xCystine; 1xZn2]	-28.6
b5 -H_2O	[1-5]	556.2072	1	.MDPET.c	-1.7
b5	[1-5]	574.2177	1	.MDPET.c	-1.0
y5	[64-68]	605.0495	1	k.CSCCQ. [2xCystine; 1xZn]	-1.5
b13	[1-13]	631.2240	2	.MDPETCPCPSGGS.c [1xCystine]	-5.4
y6 -H_2O	[63-68]	653.2204	1	e.KCSCCQ.	-0.1
b6 -H_2O	[1-6]	659.2164	1	.MDPETC.p	-1.0
y6	[63-68]	671.2310	1	e.KCSCCQ.	-1.3
b6	[1-6]	677.2269	1	.MDPETC.p	-1.1
y7	[62-68]	800.2736	1	a.EKCSCCQ.	-1.2
y8	[61-68]	871.3107	1	e.AEKCSCCQ.	-1.3
y9	[60-68]	1000.3533	1	a.EAEKCSCCQ.	-2.1
y10	[59-68]	1071.3904	1	e.AEAEKCSCCQ.	-1.3
y10	[59-68]	1133.3039	1	e.AEAEKCSCCQ. [2xCystine; 1xZn]	-1.7

Table S5. List of fragment ions identified for the CID spectrum of quadrupole-selected $apoMT3_{red}^{5+}$.

Ion	Slice	*mz*	*z*	Sequence	Error (ppm)
y11 $-H_2O$ $-NH_3$	[58-68]	388.1290	3	a.EAEAEKCSCCQ. [3xCystine]	-18.2
y4	[65-68]	440.1268	1	c.SCCQ.	19.1
b14	[1-14]	455.1522	3	.MDPETCPCPSGGSC.t [2xCystine]	-14.3
b4	[1-4]	473.1701	1	.MDPE.t	1.1
b15	[1-15]	489.1707	3	.MDPETCPCPSGGSCT.c [1xCystine]	5.5
b10	[1-10]	531.1905	2	.MDPETCPCPS.g	-19.9
y5	[64-68]	543.1360	1	k.CSCCQ.	2.3
b5 $-H_2O$	[1-5]	556.2072	1	.MDPET.c	-6.5
b5	[1-5]	574.2177	1	.MDPET.c	5.3
y19 $-H_2O$	[50-68]	633.2564	3	c.VCKGGEAAEAEAEKCSCCQ.	-2.5
b6 $-H_2O$	[1-6]	659.2164	1	.MDPETC.p	2.2
b6	[1-6]	677.2269	1	.MDPETC.p	-1.4
y17 $-H_2O$ $-NH_3$	[52-68]	839.8289	2	c.KGGEAAEAEAEKCSCCQ.	-5.1
y17	[52-68]	857.3475	2	c.KGGEAAEAEAEKCSCCQ.	-8.7
b8	[1-8]	877.2889	1	.MDPETCPC.p	6.9
y21	[48-68]	1067.4043	2	k.DCVCKGGEAAEAEAEKCSCCQ.	-9.4
y10	[59-68]	1068.3669	1	e.AEAEKCSCCQ. [3xCystine]	12.4
y20	[49-68]	1008.8840	2	d.CVCKGGEAAEAEAEKCSCCQ. [2xCystine]	5.0
y7	[62-68]	800.2736	1	a.EKCSCCQ.	0.4
y9	[60-68]	1000.3533	1	a.EAEKCSCCQ.	18.1
y10	[59-68]	1069.3747	1	e.AEAEKCSCCQ. [2xCystine]	8.0
y10	[59-68]	1070.3826	1	e.AEAEKCSCCQ. [1xCystine]	15.0
y10	[59-68]	1071.3904	1	e.AEAEKCSCCQ.	6.0
y30	[39-68]	1031.0736	3	c.PAECEKCAKDCVCKGGEAAEAEAEKCSCCQ. [2xCystine]	7.6

Table S6. List of fragment ions identified for the CID spectrum of quadrupole-selected $Cu(I)_4MT3_{ox}^{5+}$.

Ion	Slice	*mz*	*z*	Sequence	Error (ppm)
b3	[1-3]	344.1275	1	.MDP.e	9.6
y3	[66-68]	351.0791	1	s.CCQ. [2xCystine]	9
y3	[66-68]	352.087	1	s.CCQ. [1xCystine]	-3.6
y3	[66-68]	353.0948	1	s.CCQ.	6.2
y4	[65-68]	440.1268	1	c.SCCQ.	4.1
b4 -H_2O	[1-4]	455.1595	1	.MDPE.t	-3.4
b4	[1-4]	473.1701	1	.MDPE.t	0.2
y9 -H_2O	[60-68]	491.1711	2	a.EAEKCSCCQ. [1xCystine]	12
y9	[60-68]	500.1764	2	a.EAEKCSCCQ. [1xCystine]	19.1
y9	[60-68]	500.6803	2	a.EAEKCSCCQ.	-4.1
y5 -H_2O	[64-68]	523.1098	1	k.CSCCQ. [2xCystine]	-7.9
y5 -H_2O	[64-68]	524.1176	1	k.CSCCQ. [1xCystine]	-4.5
y5 -H_2O	[64-68]	525.1254	1	k.CSCCQ.	-2.9
y5 -NH_3	[64-68]	526.1095	1	k.CSCCQ.	-8.5
y10	[59-68]	536.1988	2	e.AEAEKCSCCQ.	-5.5
y5	[64-68]	541.1204	1	k.CSCCQ. [2xCystine]	-0.5
y5	[64-68]	543.136	1	k.CSCCQ.	0.2
b5 -H_2O	[1-5]	556.2072	1	.MDPET.c	-1
b5	[1-5]	574.2177	1	.MDPET.c	0.8
b6 -H_2O	[1-6]	659.2164	1	.MDPETC.p	0.4
y6	[63-68]	669.2153	1	e.KCSCCQ. [2xCystine]	-1.2
y6	[63-68]	671.231	1	e.KCSCCQ.	-1.2
b6	[1-6]	677.2269	1	.MDPETC.p	0.3
y7 -H_2O	[62-68]	782.263	1	a.EKCSCCQ.	2.9
y7	[62-68]	798.2579	1	a.EKCSCCQ. [2xCystine]	7.4
y7	[62-68]	799.2657	1	a.EKCSCCQ. [1xCystine]	0.6
y7	[62-68]	800.2736	1	a.EKCSCCQ.	6.5

y25	[44-68]	854.6788	3	e.KCAKDCVCKGGEAAEAEAEKCSCCQ. [2xCystine]	9.4
y25	[44-68]	855.3507	3	e.KCAKDCVCKGGEAAEAEAEKCSCCQ.	7.8
y17	[52-68]	856.3397	2	c.KGGEAAEAEAEKCSCCQ. [2xCystine]	12.2
y17	[52-68]	857.3475	2	c.KGGEAAEAEAEKCSCCQ.	10.7
y8	[61-68]	869.295	1	e.AEKCSCCQ. [2xCystine]	11.4
y8	[61-68]	871.3107	1	e.AEKCSCCQ.	10.6
b8	[1-8]	877.2889	1	.MDPETCPC.p	8.1
y18	[51-68]	907.8442	2	v.CKGGEAAEAEAEKCSCCQ. [2xCystine]	13.9
y18	[51-68]	908.8521	2	v.CKGGEAAEAEAEKCSCCQ.	11.2
y19	[50-68]	957.3785	2	c.VCKGGEAAEAEAEKCSCCQ. [2xCystine]	-16.1
y19	[50-68]	958.3863	2	c.VCKGGEAAEAEAEKCSCCQ.	12.2
y9	[60-68]	1000.3533	1	a.EAEKCSCCQ.	18.6
y20	[49-68]	1009.8909	2	d.CVCKGGEAAEAEAEKCSCCQ.	16.8
y30	[39-68]	1031.0736	3	c.PAECEKCAKDCVCKGGEAAEAEAEKCSCCQ. [2xCystine]	19.3
y30	[39-68]	1031.7455	3	c.PAECEKCAKDCVCKGGEAAEAEAEKCSCCQ.	15.3
y10	[59-68]	1069.3747	1	e.AEAEKCSCCQ. [2xCystine]	5.2
y10	[59-68]	1070.3826	1	e.AEAEKCSCCQ. [1xCystine]	7.9
y10	[59-68]	1071.3904	1	e.AEAEKCSCCQ.	2.4

REFERENCES

1 Krężel, A.; Maret, W. The bioinorganic chemistry of mammalian metallothioneins. *Chem. Rev.* **2021**, *121*, 14594–14648.

2 Eyer, P.; Worek, F.; Kiderlen, D.; Sinko, G.; Stuglin, A.; Simeon-Rudolf, V.; Reiner, E. Molar absorption coefficients for the reduced Ellman reagent: reassessment. *Anal. Biochem.* **2003**, *312*, 224–227.

3 Peris-Díaz, M. D.; Guran, R.; Domene, C.; de los Rios, V.; Zitka, O.; Adam, V.; Krężel, A. An integrated mass spectrometry and molecular dynamics simulations approach reveals the spatial organization impact of metal-binding sites on the stability of metal-depleted metallothionein-2 species. *J. Am. Chem. Soc.* **2021**, *143*, 16486–16501.

4 Peris-Díaz, M. D.; Guran, R..; Zitka, O.; Adam, V.; Krężel, A. Metal- and affinity-specific dual labeling of cysteine-rich proteins for identification of metal-binding sites. *Anal. Chem.* **2020**, *92*, 12950–12958.

5 France, A. P.; Migas, L. G.; Sinclair, E.; Bellina, B.; Barran, P. E. Using collision cross section distributions to assess the distribution of collision cross section values. *Anal. Chem.* **2020**, *92*, 4340–4348.

6 Migas, L. G.; France, A. P.; Bellina, B.; Barran, P. E. ORIGAMI: a software suite for activated ion mobility mass spectrometry (aIM-MS) applied to multimeric protein assemblies. *Int. J. Mass Spectrom.* **2018**, *427*, 20–28.

7 Gabelica, V.; DePauw, E. Internal energy and fragmentation of ions produced in electrospray sources. *Mass Spectrom. Rev.* **2005**, *25*, 566–587.

8 Virtanen, P. et al. SciPy 1.0: Fundamental algorithms for scientific computing in python. *Nat. Methods*, **2020**, *17*, 261–272.

9 Strohalm, M.; Kavan, D.; Novák, P.; Volný, M.; Havlíček, V. mMass 3: a cross-platform software environment for precise analysis of mass spectrometric data. *Anal. Chem.* **2010**, *82*, 4648–4651.
